# The Integration of Genome Mining, Comparative Genomics, and Functional Genetics for Biosynthetic Gene Cluster Identification

**DOI:** 10.3389/fgene.2020.600116

**Published:** 2020-12-03

**Authors:** Ashley N. Williams, Naveen Sorout, Alexander J. Cameron, John Stavrinides

**Affiliations:** Department of Biology, University of Regina, Regina, SK, Canada

**Keywords:** *Pantoea*, secondary metabolites, biosynthetic gene cluster, agar overlay assay, genome mining, antiSMASH, comparative genomics, EDGAR 2

## Abstract

Antimicrobial resistance is a worldwide health crisis for which new antibiotics are needed. One strategy for antibiotic discovery is identifying unique antibiotic biosynthetic gene clusters that may produce novel compounds. The aim of this study was to demonstrate how an integrated approach that combines genome mining, comparative genomics, and functional genetics can be used to successfully identify novel biosynthetic gene clusters that produce antimicrobial natural products. Secondary metabolite clusters of an antibiotic producer are first predicted using genome mining tools, generating a list of candidates. Comparative genomic approaches are then used to identify gene suites present in the antibiotic producer that are absent in closely related non-producers. Gene sets that are common to the two lists represent leading candidates, which can then be confirmed using functional genetics approaches. To validate this strategy, we identified the genes responsible for antibiotic production in *Pantoea agglomerans* B025670, a strain identified in a large-scale bioactivity survey. The genome of B025670 was first mined with antiSMASH, which identified 24 candidate regions. We then used the comparative genomics platform, EDGAR, to identify genes unique to B025670 that were not present in closely related strains with contrasting antibiotic production profiles. The candidate lists generated by antiSMASH and EDGAR were compared with standalone BLAST. Among the common regions was a 14 kb cluster consisting of 14 genes with predicted enzymatic, transport, and unknown functions. Site-directed mutagenesis of the gene cluster resulted in a reduction in antimicrobial activity, suggesting involvement in antibiotic production. An integrated approach that combines genome mining, comparative genomics, and functional genetics yields a powerful, yet simple strategy for identifying potentially novel antibiotics.

## Introduction

Antimicrobial resistance remains one of the greatest health threats worldwide (Aslam et al., 2018). Increased resistance, combined with limited investment in antibiotic discovery and development, has left healthcare providers with few options to treat multi-drug resistant bacterial infections (Aslam et al., 2018; Morehead and Scarbrough, 2018). In 2017, in response to the growing number of drug-resistant bacteria, the World Health Organization released a list of pathogens for which there are few treatment options (World Health Organization, 2017). This included the Gram-negative species *Acinetobacter baumannii*, *Pseudomonas aeruginosa*, *Salmonella* sp., and other members of the *Enterobacteriaceae*, as well as Gram-positive species *Enterococcus faecium* and *Staphylococcus aureus*. While many promising antibiotics have entered clinical trials in recent years, the majority of these antibiotics are from existing classes of therapeutics, and may eventually become ineffective due to existing resistance determinants (Hutchings et al., 2019). Furthermore, the majority of approved antibiotics in the past decade have been ineffective against pathogens of greatest concern, such as carbapenem-resistant Gram-negative bacteria (Moss and Boucher, 2020), highlighting the importance of identifying additional unique bioactive molecules.

Natural products remain a promising source of novel therapeutics (Katz and Baltz, 2016). Many pharmaceuticals have been derived from the secondary metabolites of bacteria and fungi with a variety of clinical applications, including cardiovascular drugs, chemotherapeutics, immunomodulators, and antibiotics (Patridge et al., 2016). However, the exploration of bioactive natural product antibiotics has been limited to a relatively small pool of genera, including the bacterial genus, *Streptomyces* and the fungal genera, *Penicillium* and *Cephalosporium* (Patridge et al., 2016). Since the discovery of streptomycin in *Streptomyces griseus* in 1944, actinomycetes, namely *Streptomyces*, have been the primary source of antibiotics (Watve et al., 2001; de Lima Procópio et al., 2012). Nonetheless, many species outside of the Actinobacteria have also been identified as antimicrobial producers (Fischbach, 2009; Pidot et al., 2014; Hutchings et al., 2019).

One antibiotic-producing group is the bacterial genus *Pantoea*, which belongs to the *Erwiniaceae* of the Enterobacterales (Adeolu et al., 2016). *Pantoea* antimicrobials have been predominantly explored for their antibiosis toward agricultural pathogens, such as *Erwinia amylovora* the causative agent of fire blight in apple and pear crops (Johnson and Stockwell, 1998; Pusey, 2002; Stockwell et al., 2002; Walterson and Stavrinides, 2015). Among the more well-studied antibiotics effective against *E. amylovora* are *Pantoea* Natural Product 1 (identified as 4-formylaminooxyvinylglycine; PNP-1) (Walterson et al., 2014; Okrent et al., 2018; Smith et al., 2019), pantocin A (Wright et al., 2001; Jin et al., 2003a; Smits et al., 2019), pantocin B (Brady et al., 1999; Wright et al., 2001), and herbicolin I/dapdiamide E (Ishimaru et al., 1988; Sammer et al., 2009, 2012; Dawlaty et al., 2010; Kamber et al., 2012). Comparatively, fewer *Pantoea* natural products have been investigated for potential antagonism toward clinical pathogens. Andrimid, a well-studied antibiotic, is a hybrid non-ribosomal peptide-polyketide that exhibits broad-spectrum activity against many clinically relevant bacteria including *Enterococcus*, *Staphylococcus*, and several genera within the *Enterobacteriaceae*. (Needham et al., 1994; Singh et al., 1997; Sánchez et al., 2013; Matilla et al., 2016). Andrimid, which functions by inhibiting acetyl-CoA carboxylase (Freiberg et al., 2004), is not only produced by *Pantoea* (Jin et al., 2006), but also strains of *Pseudomonas fluorescens* (Needham et al., 1994; Singh et al., 1997), *Vibrio* sp. (Long et al., 2005; Wietz et al., 2010), and *Serratia* sp. (Matilla et al., 2016). More recently, the two antibiotics *Pantoea* Natural Product 2 (PNP-2) and *Pantoea* Natural Product 3 (PNP-3) have been shown to be effective against several clinical pathogens. PNP-2 exhibits antimicrobial activity against several genera within the *Enterobacteriaceae* and *Erwiniaceae*, as well as *S. aureus* (Robinson et al., 2020). PNP-3 inhibits growth of multi-drug resistant *A. baumannii*, *P. aeruginosa*, multiple genera of the *Enterobacteriaceae*, as well as *S. aureus* and *Streptococcus mutans* (Williams and Stavrinides, 2020). This suggests that further exploring the metabolic diversity of *Pantoea* species could yield potentially novel antimicrobial metabolites.

One strategy for bioprospecting for antimicrobials involves the identification of biosynthetic gene clusters. A biosynthetic gene cluster is a modular unit of two or more contiguous genes that are responsible for metabolite production (Medema et al., 2015). These genes encode proteins that synthesize the final product, and often include genes encoding regulatory elements, transport proteins, resistance factors, or those involved in precursor production (Cimermancic et al., 2014; Tietz and Mitchell, 2015). Identifying gene clusters that direct the biosynthesis of new antimicrobials may not only facilitate the identification of novel compounds, but also can provide the means for metabolite production through heterologous expression (Medema et al., 2015; Huo et al., 2019). Identification of these biosynthetic clusters can be achieved by surveying mutant libraries for loss of antibiotic activity (Ochman et al., 1988; Walterson et al., 2014; Robinson et al., 2020; Williams and Stavrinides, 2020), or by creating expression libraries, and surveying clones for antibiotic production (Tuan et al., 1990; Wright et al., 2001; Ziemert et al., 2016).

The genomics era has also provided genome mining tools to identify candidate antibiotic biosynthetic gene clusters (Medema and Fischbach, 2015). Algorithms that have been developed to search for secondary metabolite gene clusters include antiSMASH (Medema et al., 2011; Blin et al., 2019), BAGEL (de Jong et al., 2006; van Heel et al., 2018), NP.searcher (Li et al., 2009), and PRISM (Skinnider et al., 2015, 2017). Many of these algorithms, however, rely on patterns derived from known antibiotic gene clusters and associated protein motifs, thereby limiting *de novo* prediction. Modified strategies have been developed to attempt to predict novel biosynthetic clusters, such as mining for duplicated or altered housekeeping enzymes (e.g., ARTS) (Alanjary et al., 2017), surveying for the presence of specific resistance mechanisms (e.g., ARTS and CARD) (McArthur et al., 2013), or identifying regulators and/or regulator binding sites (e.g., CASSIS) (Wolf et al., 2016). These genomic signatures may point to the presence of novel biosynthetic gene clusters not identified with other genome mining methods (Ziemert et al., 2016).

Comparative genomics strategies can also be useful for identifying novel biosynthetic gene clusters, as they are pattern-independent strategies (Takeda et al., 2014; Hautbergue et al., 2018). Using comparative genomic strategies to identify all genes unique to a strain of interest, however, can potentially generate extensive lists of candidate genes. To attempt to reduce the number of candidate genes, strategies that combine genome mining with comparative genomics can be used. For example, one strategy used to identify the gene clusters for multiple antibiotics in fungal strains involved searching for secondary metabolite genes of interest (e.g., a terpene synthase to identify terpenoid clusters) in genomic scaffolds using tblastn (Chooi et al., 2010; Cacho et al., 2015). The number of scaffolds of interest was then further reduced by comparing the sequences to related strains with alternative antibiotic production profiles to identify regions unique to the strain of interest (Chooi et al., 2010; Cacho et al., 2015). Searching for secondary metabolite biosynthetic clusters using this approach is effective, but may be time consuming if the identity or class of secondary metabolite is unknown.

Here, we propose a simplification of this approach by combining online genome mining tools with comparative genomics to identify biosynthetic gene clusters encoding potentially novel antibiotics in bacterial genomes. Unlike other strategies, our approach involves a subtractive analysis whereby gene cluster candidates from genome mining prediction are cross-referenced to the list of unique genes in the strain of interest identified by comparative genomic analysis. Candidate gene clusters identified by these comparisons can then be evaluated for involvement in antibiotic production using genetic approaches. To demonstrate the utility of this approach, we surveyed a collection of 116 *Pantoea* strains for antibiotic production using a minimal media agar-based bioassay, and showed that 59 strains produce natural products that antagonize diverse clinically relevant pathogens. One antibiotic-producing strain, *P. agglomerans* B025670 (B025670), antagonized several Gram-negative pathogens, prompting us to pursue the genes responsible for metabolite biosynthesis. We compared the independent outputs of antiSMASH results for B025670 to a collection of genes unique to B025670 as determined by whole genome comparison to related strains. One promising gene cluster was common to both lists, and subsequent mutation of the cluster via homologous recombination led to a reduction in the zones of inhibition. We suggest that our proposed approach is a quick, simple and efficient method for identifying candidate antibiotic biosynthetic gene clusters without the need for extensive computational power or expertise.

## Materials and Methods

### Bacterial Strains and Growing Conditions

Strains used in this study are shown in Supplementary Table 1. Overnight cultures of *Pantoea* and *Erwinia* strains were grown at 30°C and all other strains were grown at 37°C with shaking at 220 RPM. All cultures were maintained on lysogeny broth (LB) (Miller; BD Biosciences Franklin Lakes, NJ, United States) agar and supplemented with gentamicin (gent; 30 μg/mL, 15 μg/mL for recombinants) when appropriate. *E. faecium* K0260810 was maintained on Bacto Brain Heart Infusion (BD Biosciences).

### Agar Overlay Assay

Agar overlay assays (adapted from Wright et al., 2001) were used to evaluate antibiotic production of *Pantoea* strains. Overnight cultures of target bacteria were pelleted and resuspended in an equal volume of 10 mM MgSO_4_. Strains with slower growth in overlays (*E. faecium* K0260810, *S. aureus* K5-4, *S. aureus* K1-7) were resuspended in half- or quarter-volumes of 10 mM MgSO_4_ to concentrate bacteria. Overlay agar was prepared with 3.2–4 mL molten 0.9% agar, 800 μL 5× glucose-asparagine solution (1 L 5× solution: 55.75 g K_2_HPO_4_, 22.5 g KH_2_PO_4_, 0.6 g MgSO_4_⋅7H_2_O, 1.5 g L-asparagine, 0.25 g nicotinic acid, and 100 g glucose), and 300 μL of the target bacteria (Wodzinski et al., 1994). This was poured over 1× *Escherichia coli* Minimal Media agar [20 mL; 1 L 1× solution: 0.25 g yeast extract, 20 mL glycerol, 4 g K_2_HPO_4_, 1.72 g KH_2_PO_4_, 0.5 g NaCl, 2.0 g (NH_4_)_2_SO_4_, 0.2 g C_6_H_5_Na_3_O_7_⋅2H_2_O, 0.002 g MgSO_4_⋅7H_2_O; agar final concentration: 1.5%] and allowed to solidify (Wright et al., 2001). *Pantoea* strains to be tested for antibiotic production (designated ‘test strains’) were cultured overnight without antibiotics, pelleted, resuspended in an equal volume of 10 mM MgSO_4_, and 5 μL spotted on the agar overlays. For survey overlays of *Enterobacter* sp. TX1, *Klebsiella* sp. B011499, *Kosakonia* sp. 12202, and *Pseudocitrobacter* sp. B012497, single colonies of test strains were streaked from agar plates onto overlays with toothpicks. *E. faecium* K0260810 overlays were tested with both *Pantoea* liquid culture and streaked colonies. Plates were incubated at 30°C for 16–48 h until the layer of target bacteria was opaque. All images were captured with an Epson Perfection V330 photo scanner at 1200 DPI, and brightness and contrast were adjusted to enhance zones of inhibition. All *Pantoea* survey strains were tested at least once and all overlays involving *P. agglomerans* B025670 were replicated at least twice.

### Secondary Metabolite and Comparative Genomic Analysis

Secondary metabolite profiles for 31 *Pantoea* species were generated using antiSMASH 5.1.2 (Blin et al., 2019). FASTA files of draft genomes (designated in Supplementary Table 1) were analyzed with the web version with both ‘relaxed’ and ‘loose’ detection strictness with the switches ‘-KnownClusterBlast,’ ‘-ClusterBlast,’ and ‘-SubClusterBlast.’ FASTA files of contigs containing PNP-1 (*Pantoea ananatis* BRT175), PNP-2 (*P. agglomerans* TX10), PNP-3 (*P. agglomerans* SN01080, 3581), pantocin A (*P. agglomerans* 3581), and cluster 675 (*P. agglomerans* B025670) were also analyzed with the web versions of BAGEL4 (van Heel et al., 2018), NP.Searcher (range 1–5,000; ‘-display unknown peptide or ketide units’ and ‘-none’ switches selected) (Li et al., 2009), and PRISM (all advanced settings switches on; structure limit = 50; window = 10,000 bp) (Skinnider et al., 2017). Antibiotic cluster accession numbers: BRT175 PNP-1 (ASJH00000000) (Smith et al., 2013), TX10 PNP-2 (MN329808), SN01080 PNP-3 (MN807450), 3581 PNP-3 (MN807451), B025670 675 (MT711882), and 3581 pantocin A (MT711881).

Genome comparisons of B025670 to other *Pantoea* strains were performed with EDGAR 2 (Blom et al., 2009, 2016). Genomes in EDGAR were annotated with Prokka 1.12 (Seemann, 2014). B025670 was compared to *P. agglomerans* DC432 and SP04022 (Trial 1), as well as *P. agglomerans* DC432, *P. agglomerans* SP04022, *Pantoea dispersa* 625, *Pantoea stewartii* 626, *Pantoea eucalypti* B011489, *Pantoea brenneri* B016381, *P. brenneri* B024858, *P. eucalypti* F9026, *P. ananatis* 15320, *P. ananatis* 17671, and *P. ananatis* 26SR6 (Trial 2). Nucleotide FASTA files of genes unique to B025670 and of antiSMASH regions were compared with standalone BLAST and parsed. Candidate cluster 675 was annotated with PATRIC with the following parameters: domain = bacteria; taxonomy name = *Pantoea agglomerans* (taxonomy ID = 549); genetic code = 11 (Archaea and Bacteria), and annotation recipe = default (Brettin et al., 2015). The complete nucleotide sequence of cluster 675 was searched for against the NCBI Nucleotide Collection and WGS (limit: *Pantoea*) databases with blastn. Amino acid sequences were analyzed with CDD batch search against CDD v3.18 at an *E*-value threshold of 0.01 with composition-based statistics adjustment “on.” Translated nucleotide BLASTs (blastx) were performed against the nr database, and the best hit for each gene with the lowest *E*-value and highest percent identity was recorded. Cluster 675 from *P. dispersa* M1657A was annotated with GeneMark.hmm prokaryotic v3.25 (Zhu et al., 2010). Cluster figures were generated with Easyfig 2.2.3 (Sullivan et al., 2011).

### Mutagenesis

Genes 4 and 7 of cluster 675 in B025670 were disrupted via single-crossover homologous recombination. *Taq* DNA Polymerase (GeneDireX) was used to amplify an internal fragment of these genes from B025670 using specific primers (Supplementary Table 2). Fragments were digested for 2 h with *Pst*I or *Xma*I (New England Biolabs) and cloned into the *Pst*I or *Xma*I restriction site of pKNOCKGm. Plasmid and insert were ligated in a 1:3 ratio, respectively, in a 10 μL reaction with T4 DNA ligase (New England Biolabs). The ligated product (5 μL) was electroporated into 100 μL of electrocompetent *E. coli* CC118 cells using a micropulser (Bio-Rad) at 2.5 kV in a 1 mm cuvette, and cells were plated on LB-gent agar. The transformants were confirmed by colony PCR with *Taq* DNA Polymerase (GeneDireX) using primers gent+391 and gent-57 (Supplementary Table 2). Transformants were also confirmed by PCR with the fragment-specific primers (Supplementary Table 2). Plasmids were electrotransformed into electrocompetent B025670 and plated on LB-gent agar. Integration of the plasmid was confirmed by colony PCR with *Taq* DNA Polymerase (GeneDireX) using primers gent+391 and 675_9-790_XmaI for 675-7 and gent+391 and 675_4+1 for 675-4 (Supplementary Table 2). An integration mutant of a separate gene cluster (697-2::Gm) was generated, confirmed with primers 697_1+145_PstI and gent+391, and used as a gentamicin control strain. The resulting mutants were evaluated for loss of antibiotic production on overlays of strains highlighted in Table 2. Test strains were adjusted to the same OD_600_, and non-standardized relative OD_600_ values were obtained with the Gen5 software suite (BioTek) and Epoch Microplate Spectrophotometer in a CELLSTAR 96 well plate (Greiner Bio-One) containing 300 μL culture per well.

## Results

### *Pantoea* Strains Exhibit Antimicrobial Activity Against Several Clinical Pathogens

*Pantoea* strains (116 total) were surveyed for antibiotic production against both Gram-negative (*A. baumannii*, *E. coli*, *Enterobacter* sp., *E. amylovora*, *Klebsiella* sp., *Kosakonia* sp., *P. aeruginosa*, *Pseudocitrobacter* sp., and *S. enterica* Typhimurium) and Gram-positive species (*Lactococcus lactis*, *S. aureus*, and *E. faecium*) (Supplementary Table 3). Thirty-two *Pantoea* strains antagonized either *S. aureus* K1-7 or *E. amylovora* Ea321 (Table 1), as indicated by the formation of a zone of inhibition. Between 10 and 20 *Pantoea* strains were effective against *L. lactis*, *Salmonella enterica*, *Enterobacter*, *Kosakonia*, *Pseudocitrobacter*, *E. faecium*, and *E. coli*. However, only two *Pantoea* strains (*P. agglomerans* SN01080r and 3581r) were effective against *P. aeruginosa*, *A. baumannii*, and *Klebsiella*. The range of pathogens inhibited varied across strains of *Pantoea*. For example, 59 of the 116 strains (50.9%) antagonized at least one pathogen, with 36 of the 116 strains (31.0%) antagonizing more than one pathogen (Supplementary Table 4). The remaining 57 strains did not antagonize any of the target pathogens. In addition, 39 of the 116 strains (33.6%) antagonized at least one Gram-negative strain or one Gram-positive strain, and 19 of the 116 strains (16.4%) antagonized both Gram-positive and Gram-negative strains.

**TABLE 1:** Summary of pathogen susceptibilities.

Pathogen	Number of *Pantoea* strains tested	Number of *Pantoea* strains effective	% effective
*A. baumannii* ATCC 17978	97	2	2.1
*Enterobacter sp.* TX1	96	14	14.6
*E. amylovora* Ea321	109	32	29.4
*E. coli* HB101 (RK600)	109	10	9.2
*Klebsiella* sp. B011499	96	2	2.1
*Kosakonia* sp. 12202	96	14	14.6
*Pseudocitrobacter* sp. B012497	96	13	13.5
*P. aeruginosa* ATCC 27853	109	2	1.8
*S. enterica* ATCC 14028	113	18	15.9
*E. faecium* K0260810	96	11	11.5
*L. lactis* HD1	45	20	44.4
*S. aureus* K1-7	109	32	29.4

There were also differences in antagonistic capabilities across the different *Pantoea* species groups. Over 50% of the representatives of *P. agglomerans*, *Pantoea anthophila*, *Pantoea conspicua*, and *P. dispersa* exhibited antimicrobial activity, whereas less than 50% of *P. ananatis*, *P. brenneri*, *P. eucalypti*, *Pantoea eucrina*, and *Pantoea septica* strains exhibited antibiosis (Supplementary Table 4). No strains of *Pantoea latae* and *P. stewartii* had antimicrobial activity, although these species groups were represented by only two strains each. Representative strains of *P. ananatis*, *P. conspicua*, and *P. eucrina* antagonized only Gram-negative bacteria, while those of *P. anthophila* and *P. septica* were active against only Gram-positive bacteria. Strains of *P. agglomerans*, *P. brenneri*, *P. dispersa*, and *P. eucalypti* antagonized both groups of pathogens. Strains exhibiting inhibitory activity against a single pathogen were found across multiple species groups (Supplementary Tables 3, 5). *Pantoea* strains that antagonized over 50% of the target pathogens included SN01080r, which had the broadest antibacterial activity at 11 pathogens, and DC434, 3581r, SP05120, SP01202, SP05061, EH318, TX10, B025670, M1657B, B026440, and SP03412 that antagonized between 4 and 8 pathogens each (Supplementary Tables 3, 5). These *Pantoea* strains, with the exception of TX10, B025670, M1657B, and B026440, were isolated from the environment. Strains effective against 50% or more of both Gram-negative and Gram-positive pathogens targeted included *P. agglomerans* SN01080r, SP01202, DC434, SP05120, TX10, SP03412, and *P. dispersa* M1657B (Supplementary Table 5).

### *P. agglomerans* B025670 Antagonizes Several Clinical Enterobacterales Strains

One strain of interest that emerged from the antibiotic survey was *P. agglomerans* B025670 (B025670), a clinical isolate that was able to antagonize *Enterobacter*, *E. amylovora*, *E. coli*, *Kosakonia*, *Pseudocitrobacter* sp., and *S. enterica* (Table 2 and Supplementary Table 3). Strains with similar antibiotic production profiles included *P. ananatis* BRT175 and *P. dispersa* M1657A; however, these were not shown to be effective against *E. coli* HB101 and *Pseudocitrobacter* sp. B012497 under the tested conditions. *P. dispersa* M1657B also had a similar antibiotic profile, although it lacked inhibitory activity against *E. coli* and was inhibitory against *S. aureus*. The inhibitory activity of B025670 was assayed against additional clinical isolates of *Pseudocitrobacter* sp., *E. coli*, *Enterobacter* sp., and *Klebsiella* sp. and was found to antagonize six total additional strains of *E. coli* and *Enterobacter* sp. (Supplementary Figure 1 and Table 2), including multi-drug resistant *E. coli* A6152, which is resistant to ampicillin, cephalosporins, ciprofloxacin, and gentamicin (Supplementary Table 1). The antibiosis against some strains was ambiguous, where there appeared to be reduced growth of B025670, making it difficult to assess whether a zone of inhibition was present (Supplementary Figure 1).

**TABLE 2:** Spectrum of activity of *P. agglomerans* B025670.

Pathogen	B025670 (WT)	697-2::Gm	675-4::Gm	675-7::Gm
*Acinetobacter baumannii*				
ATCC 17978	R	–	–	–
*Enterobacter hormaechei*				
ATCC 700323	S	S	R	R
*Enterobacter* sp.				
D6052	?	–	–	–
D6239	?	–	–	–
D6370	S	S	R	R
D6437	S	S	R	R
D6580	R	–	–	–
TX1	S	S	R	R
TX2	S	S	R	R
*Erwinia amylovora*				
Ea321	S	–	–	–
*Escherichia coli*				
A6112	?	–	–	–
A6141	R	–	–	–
A6151	R	–	–	–
A6152	S	S	R	R
C31C4	S	S	S	S
HB101 (RK600)	S	S	S	S
*Klebsiella pneumoniae*				
ATCC BAA 1705	R	–	–	–
*Klebsiella* sp.				
A5339	R	–	–	–
A5544	?	–	–	–
A5937	R	–	–	–
A5979	R	–	–	–
B011499	R	–	–	–
C3131	R	–	–	–
*Kosakonia* sp.				
12202	S	S	R	R
*Pseudocitrobacter* sp.				
10–854	?	–	–	–
B012497	S	S	R	R
*Pseudomonas aeruginosa*				
ATCC 27853	R	–	–	–
*Salmonella enterica* Typhimurium				
ATCC 14028	S	–	–	–
*Staphylococcus aureus*				
K1-7	R	–	–	–
K5-4	R	–	–	–

*S, susceptible; R, resistant; ?, ambiguous.*

### antiSMASH Predicts Numerous Candidate Biosynthetic Clusters

To attempt to identify the gene cluster(s) responsible for antibiotic production in B025670, antiSMASH was used to predict candidate secondary metabolite clusters (Supplementary Table 6). We also analyzed an additional 30 *Pantoea* strains with antiSMASH to provide a basis for comparison of gene cluster complements (Supplementary Table 6). The B025670 genome had 24 predicted regions, consistent with the other *Pantoea* genomes that had between 16 and 28 predicted metabolite biosynthetic clusters. Eleven of the 24 predicted B025670 gene clusters were also present in several other *Pantoea* genomes, including those involved in the biosynthesis of amonabactin P 750, aryl polyenes, carotenoids, desferrioxamine E, emulsan, herboxidiene, O&K-antigen, O-antigen, polysaccharide B, stewartan, and taxlllaid A (Supplementary Table 6). The other 13 predicted clusters of B025670, one of which we anticipated being involved in the biosynthesis of our antibiotic, included one homoserine lactone, three fatty acid, and nine saccharide gene clusters, each composed of 17–26 genes.

Genomes that carry the PNP-1, PNP-2, PNP-3, and pantocin A gene clusters were also included in our antiSMASH analysis to help provide a benchmark for the ability of antiSMASH to predict these novel gene clusters. The PNP-3 gene cluster in *P. agglomerans* SN01080, the PNP-2 cluster of *P. agglomerans* TX10, the PNP-1 cluster of *P. ananatis* BRT175, and the pantocin A cluster of *P. agglomerans* 3581 were not identified by antiSMASH (Supplementary Table 7). However, the algorithm predicted that the entire 8.5 kb PNP-3 cluster from *P. agglomerans* 3581 was part of an approximately 48 kb phosphonate/NRPS-encoding region (region 13.1; Supplementary Table 6). The PNP-1, PNP-2, PNP-3, and pantocin A gene clusters were also analyzed with genome mining tools BAGEL4, NP.searcher, and PRISM, but these tools were unable to identify the complete clusters, with the exception of pantocin A, which was correctly predicted by PRISM (Supplementary Table 7).

### Identification of Candidate Antibiotic Clusters Using Comparative Genomics

**FIGURE 1:**
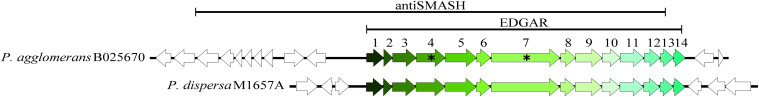
Candidate antibiotic cluster (cluster 675) of *P. agglomerans* B025670 and a homologous cluster found in *P. dispersa* M1657A (78.06% nucleotide identity, 100% query cover, *E* = 0). The lines above each region indicate the cluster prediction of antiSMASH (region 4.1), as compared to the region predicted by comparative genomic approaches using EDGAR. Asterisks denote genes that were targeted for disruption by homologous recombination. Flanking genes are shown as open (unshaded) arrows.

To narrow down the candidate list provided by antiSMASH, we used a comparative genomics approach to generate a list of genes unique to B025670 that could be responsible for antibiotic production. EDGAR identified 436 genes that were present in B025670, but absent in two closely related *P. agglomerans* strains lacking antimicrobial activity against *Enterobacter* sp. TX1 and *Kosakonia* sp. 12202. The list of unique genes was compared to the B025670 antiSMASH predictions using BLAST, and 40 genes across 5 antiSMASH regions (4.1, 18.1, 31.1, 32.2) were found to be common between the two datasets (Supplementary Table 8). Within antiSMASH regions 4.1, 18.1, 21.2, and 32.2, the majority of the common genes were contiguous and syntenic. The B025670 genome was then compared to a larger set of 11 genomes, which included more distantly related *Pantoea* strains. This comparison yielded 221 genes unique to B025670. Comparison of the gene set to the antiSMASH regions yielded 23 genes in common across four antiSMASH regions (4.1, 18.1, 21.2, 32.2) (Supplementary Table 9). Groups of sequential genes were identified in regions 4.1 (13 genes), and 32.2 (4 genes). The group of 13 genes, which was identified by both sets of comparisons, was determined to be the most likely cluster and was designated cluster 675 (Figure 1).

To delineate the boundaries of the complete cluster, a standalone BLAST of the genes and the surrounding genomic region was performed against our collection of *Pantoea* genomes. An identical cluster was identified in the clinical isolate *P. dispersa* M1675A (78% nucleotide identity, 100% query cover, *E* = 0); however, a comparison of the cluster and its flanking region indicated that the conserved portion was 14 genes and approximately 14 kb in length, the entirety of which was found in the EDGAR datasets (Figure 1). We then examined the distribution of the nucleotide sequence across bacteria in the public databases. The cluster was found in its entirety in four additional *Pantoea* draft genomes: *Pantoea* sp. EKM22T (JAALFX010000004.1, 99.54% identity, 100% query cover, *E* = 0), *Pantoea* sp. EKM21T (JAALFV010000007.1, 99.54% identity, 100% query cover, *E* = 0), *P. agglomerans* DAPP-PG734 (JNVA01000020.1, 99.52% identity, 99% query cover, *E* = 0), and *Pantoea deleyi* LMG 24200 (MIPO01000022.1; 99.18% identity, 100% query cover, *E* = 0). Cluster 675 was also analyzed with BAGEL4, NP.searcher, and PRISM, but these algorithms did not identify the region as a metabolite biosynthetic gene cluster (Supplementary Table 7).

### Characterization of Cluster 675

As cluster 675 was the leading biosynthetic gene cluster responsible for antibiotic production in B025670, its genetic composition was characterized further. The individual cluster proteins were analyzed with the CDD and BLAST and were found to include predicted enzymes involved in modification and transport (Table 3). Genes 1, 4, 5, 6, 8, 9, 10, and 11 encode predicted reductases, synthases, ligases/synthetases, and transferases. Gene 7 encodes a protein similar to an RND multidrug efflux pump subunit, sharing 41.73% amino acid identity (96% query cover, *E* = 0) with the multispecies AcrB permease subunit (WP_001132469.1). There were no predicted domains for gene 14, although the PATRIC annotation identified gene 14 as a putative transmembrane protein. Genes 3, 12, and 13 encode hypothetical proteins lacking conserved domains.

**TABLE 3:** PATRIC, CDD, and BLAST annotations for genes in cluster 675.

		CDD				Blastx				
Gene	PATRIC annotation	Specific hit (accession)	*E*-value	Superfamily (accession)	*E*-value	Annotation	Organism (accession)	*E*-value	% ID	% QC
1	Hypothetical protein	Classical short-chain dehydrogenase/reductase (cd05233)	1.51e-30	Rossmann-fold NAD(P)^(+)^ binding proteins (cl21454)	1.51e-30	SDR family NAD(P)-dependent oxidoreductase	*Kosakonia cowanii* (WP_180344384.1)	0	100	99
2	Hypothetical protein	Phosphopantetheine attachment site (pfam00550)	2.19e-04	Phosphopantetheine attachment site (cl09936)	2.35e-0	Acyl carrier protein	Unclassified *Pantoea* (WP_167423665.1)	2e-88	100	99
3	Hypothetical protein	No conserved domains	–	–	–	Hypothetical protein	*Pantoea deleyi* (ORM83485.1)	0	99.72	99
4	Hypothetical protein	Beta-ketoacyl-acyl carrier protein synthase, type I and II (cd00834)	2.57e-109	Condensing enzymes superfamily (cl09938)	2.57e-109	Beta-ketoacyl synthase family protein	*Pantoea* (WP_050491330.1)	0	100	99
5	Hypothetical protein	Acyl-CoA synthetase/AMP-acid ligase II (COG0318)	4.74e-14	Adenylate forming domain, Class I superfamily (cl17068)	6.81e-15	AMP-binding protein	*Kosakonia cowanii* (WP_139569934.1)	0	99.57	99
6	Hypothetical protein	–	–	4′-phosphopantetheinyl transferase EntD superfamily (cl39136)	1.61e-03	Hypothetical protein	*Kosakonia cowanii* (WP_139569933.1)	2e-157	100	99
7	RND efflux system, inner membrane transporter	Multidrug efflux pump subunit AcrB (COG0841)	0	Multidrug efflux pump subunit AcrB superfamily (cl34050)	0	Efflux RND transporter permease subunit	Unclassified *Pantoea* (WP_167423668.1)	0	99.42	99
8	Hypothetical protein	Methyltransferase domain (pfam13649)	2.64e-22	*S*-adenosylmethionine-dependent methyltransferases, class I (cl17173)	2.64e-22	Class I SAM-dependent methyltransferase	*Pantoea* (WP_031591949.1)	6e-164	100	99
9	Hypothetical protein	–	–	Phenylacetate-coenzyme A ligase PaaK, adenylate-forming domain family (cl34300)	1.66e-08	AMP-binding protein	Unclassified *Pantoea* (WP_167423669.1)	0	99.75	99
10	Hypothetical protein	–	–	Pyridine nucleotide-disulphide oxidoreductase (cl39093)	7.12e-04	Hypothetical protein	Unclassified *Pantoea* (WP_167423670.1)	0	100	99
11	Hypothetical protein	–	–	Citrate synthase (cl29032)	7.23e-11	Hypothetical protein	*Pantoea agglomerans* (WP_031591946.1)	0	99.72	99
12	Hypothetical protein	No conserved domains	–	–	–	Hypothetical protein	Unclassified *Pantoea* (WP_167423672.1)	5e-152	99.17	99
13	Hypothetical protein	No conserved domains	–	–	–	Hypothetical protein	*Pantoea* (WP_050491328.1)	4e-124	100	99
14	Putative transmembrane protein	No conserved domains	–	–	–	Hypothetical protein	*Pantoea deleyi* (WP_128084789.1)	1e-79	98.83	99

*QC, query cover; ID, identity.*

**FIGURE 2:**
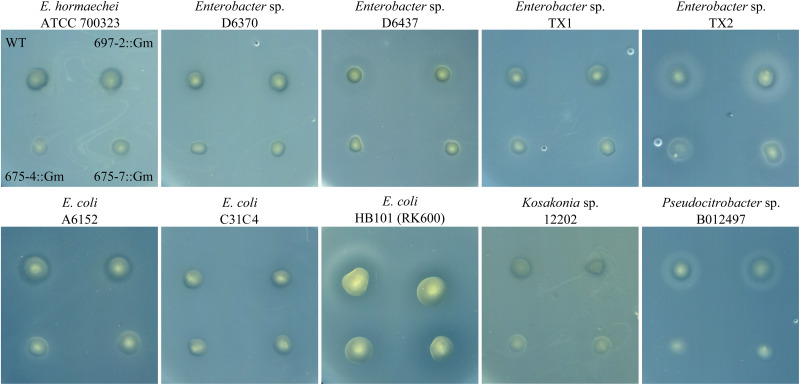
Antibiotic production assay showing an overlay of multiple target bacteria spotted with wild-type *P. agglomerans* B025670 (WT), next to a gentamicin control mutant of B025670 (697-2::Gm). The bottom set of spots on each plate show B025670 mutants with disruptions in two different genes of cluster 675 (675-4::Gm, 675-7::Gm).

To assess whether cluster 675 of B025670 was related to antibiotic production, individual genes were disrupted by single-integration homologous recombination and mutants assayed for loss of antibiotic production. Disruption of either gene 4 (beta-ketoacyl synthase) or gene 7 (RND multidrug efflux pump subunit) resulted in a loss of zone of inhibition on *Enterobacter* sp. ATCC 700323, D6370, D6437, TX1, and TX2, *E. coli* A6152, *Pseudocitrobacter* sp. B012497, and *Kosakonia* sp. 12202 overlays when compared to the wild-type strain and a gentamicin control (Figures 1, 2). Zones of inhibition were still observed for mutants tested on overlays of *E. coli* C31C4 and HB101 (Figure 2), possibly indicating the presence of additional antimicrobial metabolites.

## Discussion

In this study, we sought to identify candidate antibiotic gene clusters in bioactive *Pantoea* strains by combining genome mining, comparative genomics, and functional genetics approaches. We first identified bioactive *Pantoea* by surveying 116 strains for antimicrobial activity against a panel of pathogens on a minimal medium (Supplementary Table 3). Our use of a minimal medium was previously shown to be important for conditional expression of secondary metabolite gene clusters in *Pantoea*, including PNP-1 (Walterson et al., 2014), PNP-2 (Robinson et al., 2020), and PNP-3 (Williams and Stavrinides, 2020). This is also the case for pantocin A and B in *P. agglomerans* EH318 (Wright et al., 2001). Gene clusters that are conditionally expressed are often referred to as silent biosynthetic gene clusters, but can be activated through a variety of means, including manipulation of media composition and other growth conditions (Reen et al., 2015; Zhang et al., 2019). For PNP-1 and PNP-3, altering the composition of the top and base medium in agar overlays led to changes in the size of the zones of inhibition, and in the case of PNP-3, use of a complex medium in both layers caused complete loss of zone of inhibition (Walterson et al., 2014; Williams and Stavrinides, 2020). For pantocin A, introduction of exogenous histidine neutralizes the effects of the antibiotic on the target pathogen, and also decreases *paaA* transcription, demonstrating a link between nutrition and antibiotic expression (Wright et al., 2001; Klein et al., 2017). Therefore, the choice of medium can have a considerable impact on the observation of antimicrobial activity.

Our antibiotic production survey on a minimal medium identified 59 *Pantoea* strains exhibiting antimicrobial activity against clinically relevant genera, including *A. baumannii*, *Enterobacter*, *E. faecium*, *E. coli*, *Klebsiella*, *Kosakonia*, *Pseudocitrobacter*, *P. aeruginosa*, *S. enterica*, and *S. aureus* (Supplementary Tables 3, 4). Among the bioactive strains, 36 *Pantoea* were active against multiple pathogens, 19 exhibited activity against both Gram-negative and Gram-positive bacteria, and 23 had activity against a single target, although some of these strains were tested against a limited number of target pathogens (Supplementary Tables 3, 4). For *Pantoea* strains exhibiting antimicrobial activity against multiple pathogens, the antagonism of multiple species could be due to a single broad-spectrum antibiotic or multiple antibiotics each having narrower activity. In the case of *P. agglomerans* TX10 and 3581, multiple antibiotics, including pantocin A and their respective PNP antibiotics, were responsible for the observed spectrum of activity under the tested conditions (Robinson et al., 2020; Williams and Stavrinides, 2020). Both broad- and narrow-spectrum antibiotics have applications as therapeutics; however, as screening methods to identify causal agents of disease improve, the ability to target specific bacteria is becoming increasingly important (Melander et al., 2018).

Among the *Pantoea* strains with a more narrow-spectrum activity was *P. agglomerans* B025670 (B025670), which is effective against the Gram-negative human pathogenic species *Enterobacter* sp., *E. coli*, *Kosakonia* sp., *Pseudocitrobacter* sp., and *S. enterica* Typhimurium (Supplementary Table 3; Table 2). Of these bacteria, drug-resistant *Enterobacter* sp., *E. coli*, and *Salmonella* sp. are listed as critical and high priority pathogens for which new therapeutics are needed (World Health Organization, 2017). Therefore, B025670 was a promising strain for further exploration. The approach for identifying the B025670 antibiotic biosynthetic gene cluster involved comparing the list of antiSMASH-predicted secondary metabolite clusters of B025670 to a list of genes that were unique to B025670, but absent in strains with contrasting antibiotic profiles. Cluster 675 emerged as one of the leading candidates (Figure 1).

An analysis of the genetic composition and organization of cluster 675 revealed that it shared some characteristics with other antibiotic gene clusters including the presence of modifying enzymes such as synthases, reductases, ligases/synthetases, and transferases, as well as a transporter (Table 3). To confirm the involvement of the candidate cluster in antibiotic production, we used homologous recombination to disrupt two independent genes within the cluster, which led to a reduction in antibiotic production against several of our target bacteria (Figure 2 and Table 2). This suggests that this cluster is likely involved in the biosynthesis of an antibiotic product, or at least a necessary precursor. Whether or not the gene cluster is operonic, it is apparent that the beta-ketoacyl synthase (gene 4) and multidrug efflux pump subunit AcrB (gene 7) are involved in antibiotic production (Figure 2). Normally, beta-ketoacyl synthases are involved in elongation during fatty acid biosynthesis (Rock and Jackowski, 2002); therefore, is it possible that the synthesized antibiotic may have a fatty acid component. As for gene 7, the *E. coli* AcrB subunit is part of an RND transporter that has a wide substrate specificity (Yu et al., 2003). These tripartite efflux pumps have been implicated in multi-drug resistance in Gram-negative bacteria (Nikaido and Takatsuka, 2009), suggesting the transporter may be involved in export of the final antimicrobial product from the cell. Interestingly, the loss of the zone of inhibition for cluster 675 mutants was not observed for all the tested pathogens, suggesting the production of additional mechanisms of antibiosis by B025670 (Figure 2 and Table 2). If cluster 675 is responsible for synthesis of a precursor rather than the antibiotic itself, additional genes or gene clusters identified with the comparative genomic analysis may be involved in synthesis of the final product. Among the genes identified by the second EDGAR analysis (pre-antiSMASH comparison) were 18 clusters consisting of at least three contiguous genes (data not shown), which could be evaluated further for any possible involvement in antibiotic production. There are also additional genetic regions that are common to both the EDGAR and antiSMASH gene lists that could be explored (Supplementary Tables 8, 9).

While cross-referencing genome mining and comparative genomics data can very quickly identify candidate clusters, this strategy may not always identify the genes of interest. There may be cases where there is no overlap between datasets or identified candidates are not involved in antibiotic production. Under these circumstances, clusters and genes from individual datasets could be eliminated based on the distribution and size of typical antibiotic biosynthetic gene clusters. For example, our previous studies have shown that some *Pantoea* natural products are not widely distributed (Walterson et al., 2014; Robinson et al., 2020; Williams and Stavrinides, 2020). This is also true for the biosynthetic gene cluster of pantocin A, which was found to be present in 5 of 45 (11%) *Pantoea* strains of a variety of species (Kamber et al., 2012). In the case of B025670, several candidate clusters identified by antiSMASH could be excluded due to a wide distribution and known function, such as the cluster encoding the siderophore, desferrioxamine E, which is broadly distributed across *Pantoea* species (Supplementary Table 6) (Soutar and Stavrinides, 2018). Unlike the antibiotic gene clusters of many *Streptomyces* and other bacterial species that can exceed 100 kb (Nah et al., 2017), the conditionally-observed *Pantoea* antibiotic clusters tend to be smaller. For example, the PNP-1 cluster of *P. ananatis* BRT175 has seven genes (8.2 kb) (Walterson et al., 2014), the PNP-2 cluster of *P. agglomerans* TX10 has six genes (5.7 kb) (Robinson et al., 2020), the PNP-3 cluster of *P. agglomerans* 3581 and SN01080 has eight genes (8.5 kb) (Williams and Stavrinides, 2020), the pantocin A cluster of *P. agglomerans* EH318 has three genes plus a precursor peptide (Jin et al., 2003b), and the herbicolin I cluster of *P. vagans* C9-1 is composed of ten genes (Kamber et al., 2012). While many *Pantoea* antibiotics are the product of small biosynthetic gene clusters, some can be larger, such the cluster responsible for andrimid synthesis in *P. agglomerans* EH335 that is composed of 21 genes (Jin et al., 2006). Smaller cluster sizes can be easier to overexpress, facilitating the efficient isolation and identification of the bioactive molecule. For example, heterologous expression of several *Pantoea* antibiotics has been successful, like pantocin A, which was discovered by cosmid expression (Wright et al., 2001; Jin et al., 2003b). More recently, the PNP-2 cluster was cloned and expressed in *E. coli* (Robinson et al., 2020), while heterologous expression of the four predicted biosynthetic genes of the PNP-3 cluster in *E. amylovora* led to antibiotic production (Williams and Stavrinides, 2020). Therefore, depending on the organism of interest, antibiotic cluster size as well as distribution can be used as criteria for filtering candidate gene cluster lists.

A combinatorial approach involving genome mining, comparative genomics, and functional genetics, is a powerful method for identifying potentially novel antibiotics. On their own, genome mining approaches have considerable utility for predicting typical or known antibiotic clusters, for assigning potential function, and even predicting the final product (Kim et al., 2017). However, these computational tools have limitations, as exemplified by their inability to predict the clusters of the PNP antibiotics, and pantocin A (Supplementary Table 7). Unlike genome mining tools, the use of comparative genomics allows for the identification of all genomic content that differs between groups of genomes, irrespective of location in the genome. This removes the bias of rule-based cluster predictions, facilitating the identification of unique and unusual antibiotic clusters as well as individual genes related to antibiotic production, thereby expanding the data available for mining tool-based cluster searching (Foulston, 2019). A comparative genomics approach, however, requires that genomes of closely related strains are available to provide a reference for comparison. While these comparisons require a minimum of two genomes, the use of numerous genomes produces a more refined list of candidates. For B025670, the increase from 2 to 11 strains for comparison led to a reduction in gene number by approximately 50%. Further, comparative genomics does not indicate whether the gene products in a cluster form a functional unit, unlike genome mining tools like antiSMASH, which can predict functional units involved in the synthesis of a specific type of secondary metabolite (Medema et al., 2011). By combining comparative genomics with the output of genome mining tools, the independent limitations of these techniques can be overcome, selecting more promising candidates that can be confirmed with functional genetics approaches such as mutagenesis or heterologous expression. Additionally, by generating independent candidate lists for comparison, our strategy prevents the exclusion of potential candidate clusters that may be missed in a step-wise procedure, such as taking the output of genome mining tools and using those sequences alone in a comparative genomics analysis. Further, this protocol is not dependent upon previous knowledge about the molecule of interest and can, therefore, be used to identify biosynthetic gene clusters for unknown compounds. Overall, expanding our repertoire of antibiotics by identifying novel biosynthetic gene clusters from underexplored sources will be critical for ensuring the availability of therapeutics for treating multi-drug resistant bacteria.

## Data Availability Statement

The datasets generated for this study can be found in online repositories. The names of the repository/repositories and accession number(s) can be found in the article/Supplementary Material.

## Author Contributions

AW, NS, and AC: experiments and writing of manuscript. JS: supervision, guiding project, and writing of manuscript. All authors contributed to the article and approved the submitted version.

## Conflict of Interest

The authors declare that the research was conducted in the absence of any commercial or financial relationships that could be construed as a potential conflict of interest.
