# Supplementary material for: The Integration of Genome Mining, Comparative Genomics, and Functional Genetics for Biosynthetic Gene Cluster Identification
Source: Front Genet. 2020 Dec 3;11:600116. doi: 10.3389/fgene.2020.600116 (PMC7744662; doi:10.3389/fgene.2020.600116)
Supplement: Supplementary file 1 [file Data_Sheet_1.docx]

**Supplementary Figures and Tables**


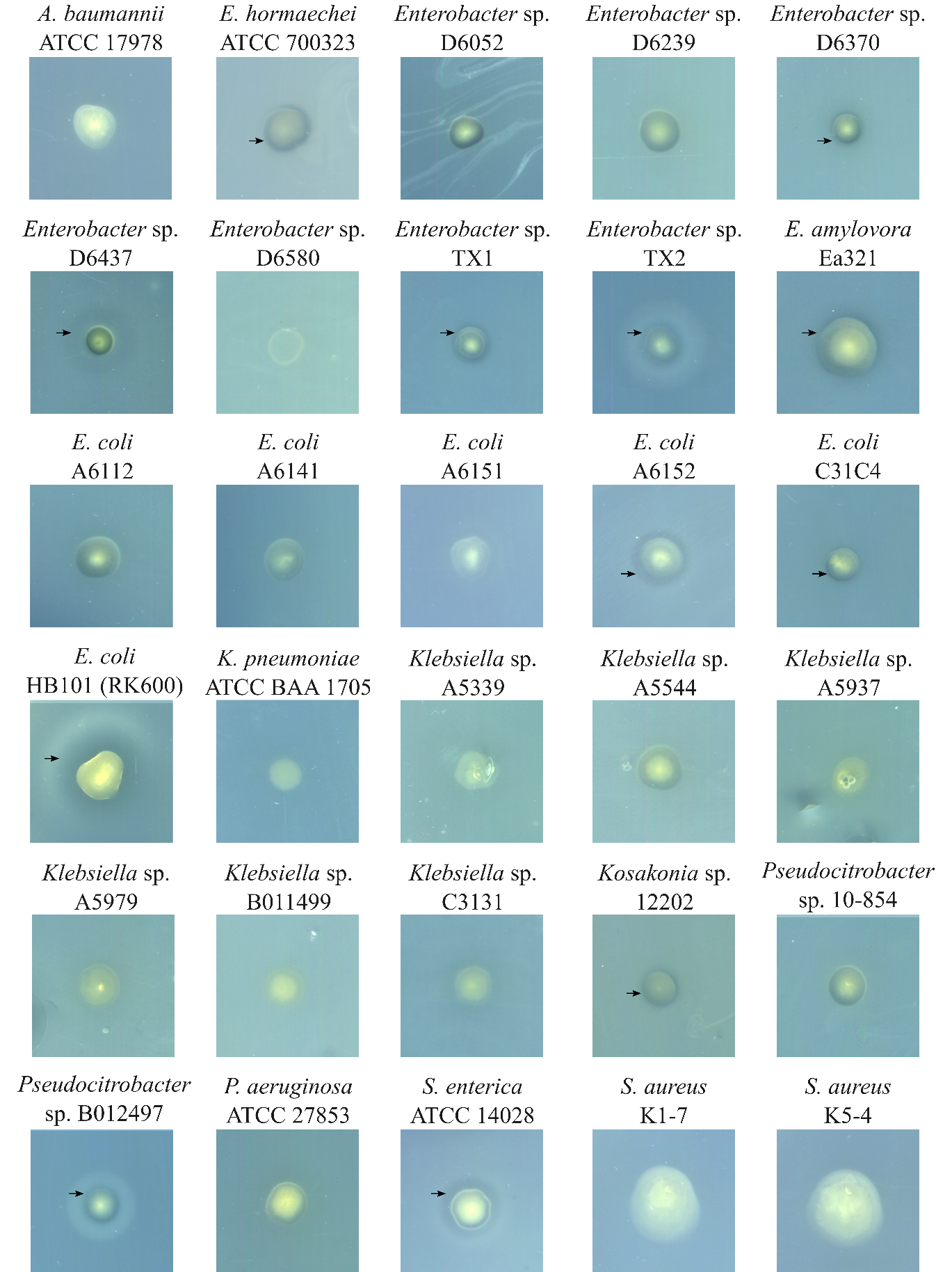


Supplementary Figure 1. Agar overlays of target bacteria spotted with *P. agglomerans* B025670. Arrows indicate borders of zones of inhibition.

Supplementary Table 1. Bacterial strains and plasmids used in this study.^a^

| Strain or plasmid | Properties | Source | Reference^b^ |
| --- | --- | --- | --- |
| *Acinetobacter baumannii* |  |  |  |
| ATCC 17978 |  | Infant with fatal meningitis | ATCC, Dr. Andrew Cameron |
| *Enterobacter hormaechei* |  |  |  |
| ATCC 700323 |  |  | ATCC, RGH |
| *Enterobacter sp.* |  |  |  |
| D6052 |  | Clinical | RGH |
| D6239 |  | Clinical | RGH |
| D6370 |  | Clinical | RGH |
| D6437 |  | Clinical | RGH |
| D6580 |  | Clinical | RGH |
| TX1 |  | Human, cystic fibrosis sputum | Texas Children’s Hospital |
| TX2 |  | Human, cystic fibrosis sputum | Texas Children’s Hospital |
| *Enterococcus faecium* |  |  |  |
| K0260810 |  | VRE-positive patient | Dr. Andrew Cameron |
| *Erwinia amylovora* |  |  |  |
| Ea321 |  | Hawthorne | ATCC |
| *Escherichia coli* |  |  |  |
| A6152 | Ampicillin (MIC ≥ 32)  Cefazolin (MIC ≥ 64)  Cefixime (MIC ≥ 4)  Cefpodoxime (MIC ≥ 8)  Ceftazidime (MIC = 4)  Ceftriaxone (MIC ≥ 64)  Gentamicin (MIC ≥ 16)  Ciprofloxacin (MIC ≥ 4) | Clinical | RGH |
| A6151 |  | Clinical | RGH |
| A6141 |  | Clinical | RGH |
| A6112 |  | Clinical | RGH |
| C31C4 |  | Clinical | RGH |
| CC118 (*λpir*) | *araD* ∆(*ara, leu*) *∆lacX74 phoA20 galE galK thi-1 rpsE rpoB argE(AM) recAl* λ*pir* |  | (Kessler et al., 1992) |
| CC118 (pKNOCKGm-675-4) | Gm^r^ |  | This study |
| CC118 (pKNOCKGm-675-7) | Gm^r^ |  | This study |
| CC118 (pKNOCKGm-697-2) | Gm^r^ |  | This study |
| HB101 (RK600) | Cm^r^, Km^s^ |  | (Kessler et al., 1992) |
| *Klebsiella pneumoniae* |  |  |  |
| ATCC BAA 1705 |  | 42-year-old male, urine | ATCC, RGH |
| *Klebsiella* sp. |  |  |  |
| A5339 |  | Clinical | RGH |
| A5544 |  | Clinical | RGH |
| A5937 |  | Clinical | RGH |
| A5979 |  | Clinical | RGH |
| B011499 |  | Human, 96-year-old female, urine indwelling catheter, cloudy urine | RRPL (Soutar and Stavrinides, 2019) |
| C3131 |  | Clinical | RGH |
| *Kosakonia* sp. |  |  |  |
| 12202 |  | Muskmelon | ICMP (Soutar and Stavrinides, 2019) |
| *Lactococcus lactis* |  |  |  |
| HD1 |  |  | Heather Dietz |
| *Pantoea agglomerans* |  |  |  |
| 22 |  | Thistle | (Soutar and Stavrinides, 2019) |
| 23 |  | Soil | (Soutar and Stavrinides, 2019) |
| 83 |  | Wheat | ICMP (Nadarasah and Stavrinides, 2014) |
| 788 |  | Green bean | ICMP (Nadarasah and Stavrinides, 2014) |
| 1512 |  | Green bean | ICMP (Nadarasah and Stavrinides, 2014) |
| 1574 |  | Unidentified | ICMP (Nadarasah and Stavrinides, 2014) |
| 3581* |  | Oat seed | ICMP (Nadarasah and Stavrinides, 2014) |
| 3581r | Rif^r^ |  | (Williams and Stavrinides, 2020) |
| 5565 |  | Soybean | ICMP (Nadarasah and Stavrinides, 2014) |
| 7373 |  | Onion | ICMP (Nadarasah and Stavrinides, 2014) |
| 7612 |  | Grass grub | ICMP (Nadarasah and Stavrinides, 2014) |
| 12531 |  | *Gypsophila* (baby’s breath) | ICMP (Nadarasah and Stavrinides, 2014) |
| 12534 |  | Human, knee laceration | ICMP (Nadarasah and Stavrinides, 2014) |
| 13301 |  | Golden delicious apple | ICMP (Nadarasah and Stavrinides, 2014) |
| 17124 |  | Olive | ICMP (Nadarasah and Stavrinides, 2014) |
| 770398 |  | Human, female, blood | Sunnybrook Hospital (Nadarasah and Stavrinides, 2014) |
| 20S |  | Bumblebee | (Soutar and Stavrinides, 2019) |
| 240R |  | Pear flower | Dr. Steven Lindow (Nadarasah and Stavrinides, 2014) |
| 308R |  | Pear flower | Dr. Steven Lindow (Nadarasah and Stavrinides, 2014) |
| B015092 |  | Human, 9-year-old female, urine midstream | RRPL (Nadarasah and Stavrinides, 2014) |
| B016395 |  | Human, 83-year-old female superficial wound | RRPL (Nadarasah and Stavrinides, 2014) |
| B025670* |  | Human, 13-year-old male, superficial wound | RRPL (Nadarasah and Stavrinides, 2014) |
| B025670 (675-4::pKNOCKGm-675-4) | B025670 derivative; homologous integration into 675-4 gene; Gm^r^ |  | This study |
| B025670 (675-7::pKNOCKGm-675-7) | B025670 derivative; homologous integration into 675-7 gene; Gm^r^ |  | This study |
| B025670 (697-2::pKNOCKGm-697-2) | B025670 derivative; homologous integration into a predicted T6SS component Hcp gene; Gm^r^ |  | This study |
| B026440 |  | Human, 10-year-old male, superficial wound | RRPL (Nadarasah and Stavrinides, 2014) |
| B23I |  | Soil | (Soutar and Stavrinides, 2019) |
| BB834250 |  | Human, female, sputum, aortic aneurysm | St. Boniface General Hospital (Nadarasah and Stavrinides, 2014) |
| DB522094 | Rif^r^ | Human, 50-year-old, elbow sore | St. Boniface General Hospital (Nadarasah and Stavrinides, 2014) |
| DC432* |  | Maize | Dr. David Coplin (Nadarasah and Stavrinides, 2014) |
| DC434 |  | Maize | Dr. David Coplin (Nadarasah and Stavrinides, 2014) |
| DC556 |  | *Gypsophila* (baby’s breath) | Dr. David Coplin (Nadarasah and Stavrinides, 2014) |
| EH318* |  | Apple leaf | CUCPB 2140; Dr. Brion Duffy (Nadarasah and Stavrinides, 2014) |
| G4032547 |  | Human, ear | RGH (Nadarasah and Stavrinides, 2014) |
| H42501 |  | Human, male, blood | Sunnybrook Hospital (Nadarasah and Stavrinides, 2014) |
| SN01080* |  | Slug | (Nadarasah and Stavrinides, 2014) |
| SN01080r | Rif^r^ |  | (Williams and Stavrinides, 2020) |
| SN01121 | Rif^r^ | Bee | (Nadarasah and Stavrinides, 2014) |
| SN01122 |  | Bee | (Nadarasah and Stavrinides, 2014) |
| SN01170 |  | Caterpillar | (Nadarasah and Stavrinides, 2014) |
| SP00101* |  | Raspberry | (Nadarasah and Stavrinides, 2014) |
| SP00202 |  | Apple | (Nadarasah and Stavrinides, 2014) |
| SP00303 |  | Raspberry | (Nadarasah and Stavrinides, 2014) |
| SP01202 |  | Strawberry leaf and stem | (Nadarasah and Stavrinides, 2014) |
| SP01220 | Rif^r^ | Healthy rose bush | (Nadarasah and Stavrinides, 2014) |
| SP01230 | Rif^r^ | Virginia creeper leaves and stem | (Nadarasah and Stavrinides, 2014) |
| SP02022 |  | Thistle | (Nadarasah and Stavrinides, 2014) |
| SP02230 |  | Diseased tree leaf | (Nadarasah and Stavrinides, 2014) |
| SP02243 |  | Unidentified tree | (Nadarasah and Stavrinides, 2014) |
| SP03310 |  | Diseased tree leaf | (Nadarasah and Stavrinides, 2014) |
| SP03383 |  | Diseased maize leaf | (Nadarasah and Stavrinides, 2014) |
| SP03412 |  | Diseased bean leaf | (Nadarasah and Stavrinides, 2014) |
| SP04010 | Rif^r^ | Tomato leaf | (Nadarasah and Stavrinides, 2014) |
| SP04011 |  | Tomato leaf | (Nadarasah and Stavrinides, 2014) |
| SP04021 |  | Tomato leaf | (Nadarasah and Stavrinides, 2014) |
| SP04022* |  | Tomato leaf | (Nadarasah and Stavrinides, 2014) |
| SP05051 |  | Tomato leaf | (Nadarasah and Stavrinides, 2014) |
| SP05052 |  | Tomato leaf | (Nadarasah and Stavrinides, 2014) |
| SP05061 | Rif^r^ | Tomato leaf | (Nadarasah and Stavrinides, 2014) |
| SP05092 |  | Tomato leaf | (Nadarasah and Stavrinides, 2014) |
| SP05120 |  | Diseased maize leaf | (Nadarasah and Stavrinides, 2014) |
| SP05130 |  | Diseased maize stamen | (Nadarasah and Stavrinides, 2014) |
| SS02010 |  | Soil, ground squirrel burrow | (Nadarasah and Stavrinides, 2014) |
| SS03231 | Rif^r^ | Soil, ground squirrel burrow | (Nadarasah and Stavrinides, 2014) |
| TX10* |  | Human, cystic fibrosis, sputum | Texas Children’s Hospital (Nadarasah and Stavrinides, 2014) |
| *Pantoea ananatis* |  |  |  |
| 15320* |  | Rice | ICMP (Nadarasah and Stavrinides, 2014) |
| 17671* |  | Rice | ICMP (Nadarasah and Stavrinides, 2014) |
| 26SR6* |  | Maize leaf | Dr. Steven Lindow (Nadarasah and Stavrinides, 2014) |
| B7* | Rif^r^ derivative of M232A | Maize | Dr. Steven Lindow (Nadarasah and Stavrinides, 2014) |
| BRT175* |  | Strawberry | Dr. Gwyn Beattie (Nadarasah and Stavrinides, 2014) |
| BRT98* | Rif^r^ | Strawberry | Dr. Steven Lindow (Nadarasah and Stavrinides, 2014) |
| Cit30-11R* | Rif^r^ | Naval orange leaf | Dr. Steven Lindow (Nadarasah and Stavrinides, 2014) |
| LMG20103 |  | Diseased eucalyptus | (De Maayer et al., 2010) |
| LMG5342 |  | Human wound | (De Maayer et al., 2012) |
| M232A* |  | Maize | Dr. Steven Lindow |
| *Pantoea anthophila* |  |  |  |
| 1373 |  | Balsam | ICMP (Nadarasah and Stavrinides, 2014) |
| *Pantoea brenneri* |  |  |  |
| 91151 |  | Human | St. Boniface General Hospital (Nadarasah and Stavrinides, 2014) |
| B011483 |  | Human, 28-year-old female, superficial wound | RRPL (Nadarasah and Stavrinides, 2014) |
| B014130 |  | Human, 11-year-old male, superficial wound | RRPL (Nadarasah and Stavrinides, 2014) |
| B016381* |  | Human, 52-year-old female, groin | RRPL (Nadarasah and Stavrinides, 2014) |
| B024858* |  | Human, 26-year-old female, breast abscess | RRPL (Nadarasah and Stavrinides, 2014) |
| *Pantoea conspicua* |  |  |  |
| B011017 |  | Human, 11-year-old female, superficial wound | RRPL (Nadarasah and Stavrinides, 2014) |
| *Pantoea dispersa* |  |  |  |
| 625* |  | Sorghum | ICMP (Nadarasah and Stavrinides, 2014) |
| M1657A* |  | Human, male, blood, morphotype 1 | Sunnybrook Hospital (Nadarasah and Stavrinides, 2014) |
| M1657B |  | Human, male, blood, morphotype 2 | Sunnybrook Hospital (Nadarasah and Stavrinides, 2014) |
| *Pantoea eucalypti* |  |  |  |
| 299R* | Rif^r^ | Pear flower | Dr. Steven Lindow (Nadarasah and Stavrinides, 2014) |
| B011489* |  | Human, 52-year-old female superficial wound | RRPL (Nadarasah and Stavrinides, 2014) |
| F9026* |  | Human, male, blood | Sunnybrook Hospital (Nadarasah and Stavrinides, 2014) |
| SM03214 |  | Goose feces | (Nadarasah and Stavrinides, 2014) |
| SP02021* | Rif^r^ | Thistle leaf | (Nadarasah and Stavrinides, 2014) |
| SP03372* |  | Diseased maize leaf | (Nadarasah and Stavrinides, 2014) |
| SP03391* |  | Diseased bean leaf | (Nadarasah and Stavrinides, 2014) |
| SP04013* |  | Tomato leaf | (Nadarasah and Stavrinides, 2014) |
| *Pantoea eucrina* |  |  |  |
| 6868 |  | Human, headache | St. Boniface General Hospital (Nadarasah and Stavrinides, 2014) |
| TX5 |  | Human, blood | Texas Children’s Hospital (Nadarasah and Stavrinides, 2014) |
| TX6 |  | Human, blood | Texas Children’s Hospital (Nadarasah and Stavrinides, 2014) |
| *Pantoea latae* |  |  |  |
| TX11 |  | Human, sputum | Texas Children’s Hospital (Soutar and Stavrinides, 2019) |
| TX7 |  | Human, blood | Texas Children’s Hospital (Soutar and Stavrinides, 2019) |
| *Pantoea septica* |  |  |  |
| 101150 |  | Human | St. Boniface General Hospital (Nadarasah and Stavrinides, 2014) |
| 062465A |  | Human, cerebellar CVA (stroke), morphotype 1 | St. Boniface General Hospital (Nadarasah and Stavrinides, 2014) |
| 062465B |  | Human, cerebellar CVA (stroke), morphotype 2 | St. Boniface General Hospital (Nadarasah and Stavrinides, 2014) |
| 091957A |  | Human, renal failure, morphotype 1 | St. Boniface General Hospital (Nadarasah and Stavrinides, 2014) |
| 091957B |  | Human, renal failure, morphotype 2 | St. Boniface General Hospital (Nadarasah and Stavrinides, 2014) |
| 81828 |  | Human, post-hemicholectomy | St. Boniface General Hospital (Nadarasah and Stavrinides, 2014) |
| B016375 |  | Human, 56-year-old female, finger | RRPL (Nadarasah and Stavrinides, 2014) |
| BB350028A |  | Human, female, blood culture, fever, morphotype 1 | St. Boniface General Hospital (Nadarasah and Stavrinides, 2014) |
| BB350028B |  | Human, female, blood culture, fever, morphotype 2 | St. Boniface General Hospital (Nadarasah and Stavrinides, 2014) |
| BE528629 |  | Human, peritoneal dialysis | St. Boniface General Hospital (Nadarasah and Stavrinides, 2014) |
| G2291404 |  | Human | RGH (Nadarasah and Stavrinides, 2014) |
| G3271436 |  | Human, urine | RGH (Nadarasah and Stavrinides, 2014) |
| G4071105 |  | Human, urine | RGH (Nadarasah and Stavrinides, 2014) |
| M1517 |  | Human, female, blood | Sunnybrook Hospital (Nadarasah and Stavrinides, 2014) |
| M41864 |  | Human, female, blood | Sunnybrook Hospital (Nadarasah and Stavrinides, 2014) |
| TX3 |  | Human, blood | Texas Children’s Hospital (Nadarasah and Stavrinides, 2014) |
| TX4 |  | Human, blood | Texas Children’s Hospital (Nadarasah and Stavrinides, 2014) |
| VB38951A* |  | Human, female, blood culture, sore throat, morphotype 1 | St. Boniface General Hospital (Nadarasah and Stavrinides, 2014) |
| VB38951B |  | Human, female, blood culture, sore throat, morphotype 2 | St. Boniface General Hospital (Nadarasah and Stavrinides, 2014) |
| X44686* |  | Human, female, blood | Sunnybrook Hospital (Nadarasah and Stavrinides, 2014) |
| *P. stewartii* |  |  |  |
| 626* |  | Maize | ICMP (Nadarasah and Stavrinides, 2014) |
| DC283* |  | Maize | Dr. David Coplin (Nadarasah and Stavrinides, 2014) |
| *Pantoea* sp. |  |  |  |
| TX9 |  | Human, foot | Texas Children’s Hospital (Soutar and Stavrinides, 2019) |
| *Pseudocitrobacter* sp. |  |  |  |
| 10-854 |  | Human, 58-year-old, abdominal fluid, ruptured appendix | (Soutar and Stavrinides, 2019) |
| B012497 | Rif^r^ | Human, 57-year-old female, urine midstream | RRPL (Soutar and Stavrinides, 2019) |
| *Pseudomonas aeruginosa* |  |  |  |
| ATCC 27853 |  | Blood culture | Heather Dietz, ATCC |
| *Salmonella enterica* Typhimurium |  |  |  |
| ATCC 14028 |  | Tissue, animal, chicken | Dr. Andrew Cameron; ATCC |
| *Staphylococcus aureus* |  |  |  |
| K1-7 |  |  | Dr. Christopher Yost |
| K5-4 |  |  | Dr. Christopher Yost |
| Plasmids |  |  |  |
| pKNOCKGm | Gm^r^; R6K *ori*, RP4 *oriT* |  | (Alexeyev, 1999) |
| pKNOCKGm-675-4 | Gm^r^; partial cluster 675 gene 4 (bases 385-807) cloned into MCS |  | This study |
| pKNOCKGm-675-7 | Gm^r^; partial cluster 675 gene 7 (bases 1297-1760) cloned into MCS |  | This study |
| pKNOCKGm-697-2 | Gm^r^; partial predicted T6SS component Hcp gene (bases 655-1149) cloned into MCS |  | This study |
| RK600 | ColE1, RK2-Mob^+^ RK2-Tra^+^, Km^s^ Cm^r^ derivative of RK2013; conjugative plasmid |  | (Kessler et al., 1992) |

^a^ATCC, American Type Culture Collection; Cm^R^, chloramphenicol resistance; Gm^R^, gentamicin resistance; ICMP, International Collection of Microorganisms from Plants; Km^s^; kanamycin sensitive; MIC, minimum inhibitory concentration; RGH, Regina General Hospital; Rif^r^, rifampicin resistance; RRPL, Roy Romanow Provincial Laboratory; VRE, vancomycin-resistant *Enterococcus*

^b^Dr. Andrew Cameron, University of Regina; Dr. Brion Duffy, Zürich University of Applied Sciences ZHAW; Dr. Christopher Yost, University of Regina; Dr. David Coplin, Ohio State University; Dr. Gwyn Beattie, Iowa State University; Dr. Steven Lindow, University of California Berkeley; Heather Dietz, University of Regina; Regina General Hospital, Regina, SK, Canada; Roy Romanow Provincial Laboratory (formerly the Saskatchewan Disease Control Laboratory), Regina, SK, Canada; St. Boniface General Hospital, Winnipeg, MB, Canada; Sunnybrook Hospital, Toronto, ON, Canada; Texas Children’s Hospital, Houston, TX, USA

*genomes used in this study

Supplementary Table 2. PCR primers used in this study.

| Primer name | Primer sequence (5' 🡪 3’)* |
| --- | --- |
| 697_1+145_PstI | GGCTGCAGAGGAGGCATTTCTGCTTGAACATC |
| 697_2+655_PstI | TTCTGCAGCACCCAGTCCGCCGTTTATATTG |
| 697_2-1149_PstI | TTCTGCAGAGGCCGGTTTCACGCTGTTAAG |
| 675_4+1 | ATGAAAAATACCTGTGATTATGAGCC |
| 675_4+385_PstI | TTCTGCAGTTTACCCGCCAGCTCAGTGATC |
| 675_4-807_PstI | TTCTGCAGGCTGCCATAGCCCAAGAGAGTAAG |
| 675_7+1297_XmaI | TCCCGGGTTGCCTCTGGACTTAGCCATTGTAC |
| 675_7-1760_XmaI | TCCCGGGCGTGAACGCTCCTGTGTCAGATAG |
| 675_9-790_XmaI | TCCCGGGCCGGTTGGATAGCGTATCAGTC |
| gent+391 | ACCGAAAAGATCAAGAGCAGC |
| gent-57 | GACGATCCCGCAGTGGCT |

* underlined portions are restriction enzyme sequences

Supplementary Table 3. Survey of antibiotic production across strains of *Pantoea*.*

| Strain | Source | *A. baumannii*  ATCC 17978^a^ | *Enterobacter*  TX1^b^ | *E. amylovora*  Ea321^a^ | *E. coli*  HB101 (RK600)*^a^* | *Klebsiella*  B011499^b^ | *Kosakonia*  12202^b^ | *Pseudocitrobacter*  B012497^b^ | *P. aeruginosa*  ATCC 27853*^a^* | *S. enterica*  ATCC 14028*^a^* | *E. faecium*  K0260810*^ab^* | *L. lactis*  HD1*^a^* | *S. aureus*  K1-7*^a^* |
| --- | --- | --- | --- | --- | --- | --- | --- | --- | --- | --- | --- | --- | --- |
| *P. agglomerans* |  |  |  |  |  |  |  |  |  |  |  |  |  |
| 22 | E |  |  |  |  |  |  |  |  | + |  |  |  |
| 23 | E |  |  |  |  |  |  |  |  | + |  |  |  |
| 83 | E | - | - | - | - | - | - | - | - | - | - | - | - |
| 788 | E | - | - | - | - | - | - | - | - | - | - | - | - |
| 1512 | E | - | - | - | - | - | - | - | - | - | - | - | - |
| 1574 | E | - | - | - | - | - | - | - | - | - | - | - | - |
| 3581r^c^ | E | + | -/+ | + | -^d^ | -/+ | + | + | -/+ | + ^d^ | - | - ^d^ | - ^d^ |
| 5565 | E | - | - | - | - | - | - | - | - | - | - | - | - |
| 7373 | E | - | + | + | - | - | + | - | - | - | - | - | - |
| 7612 | E | - | - | + | - | - | + | + | - | - | - | - | - |
| 12531 | E | - | - | - | + | - | - | - | - | + | - | - | - |
| 12534 | C | - | - | - | - | - | - | - | - | - | - | - | - |
| 13301 | E | - | - | + | - | - | - | + | - | - | + | - | + |
| 17124 | E | - | - | - | - | - | - | - | - | - | - |  | - |
| 770398 | C | - | - | - | - | - | + | + | - | + | - |  | - |
| 20S | E |  |  |  |  |  |  |  |  | + |  |  |  |
| 240R | E | - | - | - | - | - | - | - | - | - | - | - | - |
| 308R | E | - | - | - | - | - | - | - | - | - | + | - | - |
| B015092 | C | - | - | - | - | - | - | - | - | - | - |  | - |
| B016395 | C | - | - | + | - | - | - | - | - | - | - | + | + |
| B025670 | C | - | + | + | -/+ | - | + | -/+ | - | + |  |  | - |
| B026440 | C | - | + | + | - | - | + | + | - | - | - | + | + |
| B23I | E |  |  |  |  |  |  |  |  | + |  |  |  |
| BB834250 | C | - | - | + | - | - | - | - | - | - | - |  | - |
| DB522094 | C | - | - | - | - | - | - | - | - | - | - |  | - |
| DC432 | E | - | - | - | - | - | - | - | - | - | - | - | - |
| DC434 | E | - |  | + | + |  |  |  | - | + | + | + | -/+ |
| DC556 | E | - | - | - | - | - | - | - | - | - | - |  | - |
| EH318 | E | - | + | + | + | - | + | + | - | + | - |  | - |
| G4032547 | C | - | - | - | - | - | - | - | - | - | - | - | - |
| H42501 | C | - | - | - | - | - | - | - | - | - | - |  | - |
| SN01080r^c^ | E | + | **+** | + | + ^d^ | **+** | -/+ | -/+ | -/+ | + ^d^ | - | + ^d^ | + ^d^ |
| SN01121 | E | - |  | - | - |  |  |  | - | - |  | + | + |
| SN01122 | E | - | - | - | - | - | - | - | - | - | - | + | + |
| SN01170 | E | - | - | + | - | - | - | - | - | - | - | - | + |
| SP00101 | E | - |  | - | - |  |  |  | - | - |  |  | - |
| SP00202 | E | - |  | - | - |  |  |  | - | - |  | + | + |
| SP00303 | E | - |  | - | - |  |  |  | - | - |  | - | + |
| SP01202 | E | - | + | + | + | - | + | + | - | + | + |  | - |
| SP01220 | E | - |  | - | - |  |  |  | - | - |  | + | - |
| SP01230 | E | - |  | **-** | - |  |  |  | - | - |  |  | - |
| SP02022 | E |  | - | - | - | - | - | - | - | - | - | + | + |
| SP02230 | E |  | - | - | - | - | - | - | - | - | - | + | + |
| SP02243 | E | - | - | + | - | - | - | - | - | - | - | + | + |
| SP03310 | E | - | - | - | - | - | - | - | - | - | - |  | - |
| SP03383 | E |  | - | **+** | - | - | - | - | - | - | - | - | **+** |
| SP03412 | E | - | + | + | - | - | + | + | - | + | + |  | - |
| SP04010 | E | - |  | + | - |  |  |  | - | - |  | + | + |
| SP04011 | E |  | - | - | - | - | - | - | - | - | - | - | + |
| SP04021 | E | - | + | + | - | - | - | - | - | - | - | + | + |
| SP04022 | E |  | - | + | - | - | - | - | - | - | - | + | + |
| SP05051 | E | - | - | - | - | - | - | - | - | - | - | - | - |
| SP05052 | E |  | - | + | - | - | - | - | - | - | - | + | + |
| SP05061 | E | - |  | + | + |  |  |  | - | - |  | + | + |
| SP05092 | E | - | - | - | - | - | - | - | - | - | - | - | + |
| SP05120 | E | - | + | + | + | - | - | + | - | + | + | + | + |
| SP05130 | E | - | - | - | - | - | - | - | - | - | - |  | - |
| SS02010 | E | - | - | **-** | - | - | - | - | - | - | - |  | - |
| SS03231 | E | - |  | -/+ | -/+ |  |  |  | - | - |  |  | - |
| TX10^c^ | C | - | + | + | + | - | + | + | - | - | + |  | -/+ |
| *P. ananatis* |  |  |  |  |  |  |  |  |  |  |  |  |  |
| 15320 | E | - | - | - | - | - | - | - | - | - | - |  | - |
| 17671 | E | - | - | - | - | - | - | - | - | - | - |  | - |
| 26SR6 | E |  | - | - | - | - | - | - | - | - | - |  | - |
| B7 | E | - |  | + | - |  |  |  | - | - |  |  | - |
| BRT175^c^ | E | - | + | + | - | - | + | - | - | + | - | - | - |
| BRT98 | E | - | - | - | - | - | - | - | - | - | - |  | - |
| Cit30-11R | E |  | - | - | - | - | - | - | - | - | - |  | - |
| LMG20103 | E | - | - |  |  | - | - | - |  |  | - |  |  |
| LMG5342 | C | - | - |  |  | - | - | - |  |  | - |  |  |
| M232A | E | - | - | + | - | - | - | - | - | - | - |  | - |
| *P. anthophila* |  |  |  |  |  |  |  |  |  |  |  |  |  |
| 1373 | E | - | - | - | - | - | - | - | - | - | + |  | - |
| *P. brenneri* |  |  |  |  |  |  |  |  |  |  |  |  |  |
| 91151 | C | - | - | - | - | - | - | - | - | - | - |  | - |
| B011483 | C |  | - | + | - | - | - | - | - | - | - |  | - |
| B014130 | C | - | - | - | - | - | - | - | - | - | - | + | + |
| B016381 | C | - | - | - | - | - | - | - | - | - | - | - | - |
| B024858 | C | - | - | - | - | - | - | - | - | - | - |  | - |
| *P. conspicua* |  |  |  |  |  |  |  |  |  |  |  |  |  |
| B011017 | C | - | - | + | - | - | - | - | - | - | - | - | - |
| *P. dispersa* |  |  |  |  |  |  |  |  |  |  |  |  |  |
| 625 | E | - | - | - | - | - | - | - | - | - | + |  | - |
| M1657A | C | - | + | + | - | - | + | - | - | + | - |  | - |
| M1657B | C | - | + | + | - | - | + | + | - | + | - |  | + |
| *P. eucalypti* |  |  |  |  |  |  |  |  |  |  |  |  |  |
| SM03214 | E |  | - |  |  | - | - | - |  |  | + |  |  |
| 299R | E | - |  | - | - |  |  |  | - | - |  |  | - |
| B011489 | C | - | - | - | - | - | - | - | - | - | - | - | - |
| F9026 | C | - | - | - | - | - | - | - | - | - | - |  | - |
| SP02021 | E | - |  | + | - |  |  |  | - | - |  | + | + |
| SP03372 | E | - | - | - | - | - | - | - | - | - | - |  | - |
| SP03391 | E | - | - | - | - | - | - | - | - | - | + | + | + |
| SP04013 | E | - | - | - | - | - | - | - | - | - | - |  | - |
| *P. eucrina* |  |  |  |  |  |  |  |  |  |  |  |  |  |
| 6868 | C |  | - | - | - | - | - | - | - | - | - |  | - |
| TX5 | C | - | - | - | - | - | - | - | - | - | - |  | - |
| TX6 | C | - | - | - | - | - | - | - | - | + | - |  | - |
| *P. latae* |  |  |  |  |  |  |  |  |  |  |  |  |  |
| TX11 | C |  |  | - | - |  |  |  | - | - |  |  | - |
| TX7 | C |  |  | - | - |  |  |  | - | - |  |  | - |
| *P. septica* |  |  |  |  |  |  |  |  |  |  |  |  |  |
| 101150 | C | - | - | - | - | - | - | - | - | - | - |  | - |
| 062465A | C | - | - | - | - | - | - | - | - | - | - |  | + |
| 062465B | C | - | - | - | - | - | - | - | - | - | - |  | - |
| 091957A | C | - | - | - | - | - | - | - | - | - | - |  | + |
| 091957B | C | - | - | - | - | - | - | - | - | - | - |  | - |
| 81828 | C | - | - | - | - | - | - | - | - | - | - |  | - |
| B016375 | C | - | - | - | - | - | - | - | - | - | - |  | - |
| BB350028A | C | - | - | - | - | - | - | - | - | - | - |  | + |
| BB350028B | C | - | - | - | - | - | - | - | - | - | - |  | + |
| BE528629 | C | - | - | - | - | - | - | - | - | - | - |  | - |
| G2291404 | C | - | - | - | - | - | - | - | - | - | - |  | - |
| G3271436 | C | - | - | - | - | - | - | - | - | - | - |  | + |
| G4071105 | C | - | - | - | - | - | - | - | - | - | - |  | - |
| M1517 | C | - | - | - | - | - | - | - | - | - | - |  | - |
| M41864 | C | - | - | - | - | - | - | - | - | - | - |  | - |
| TX3 | C | - | - | - | - | - | - | - | - | - | - |  | - |
| TX4 | C | - | - | - | - | - | - | - | - | - | - |  | - |
| VB38951A | C | - | - | - | - | - | - | - | - | - | - |  | - |
| VB38951B | C |  | - | - | - | - | - | - | - | - | - |  | - |
| X44686 | C | - | - | - | - | - | - | - | - | - | - |  | - |
| *P. stewartii* |  |  |  |  |  |  |  |  |  |  |  |  |  |
| 626 | E | - | - | - | - | - | - | - | - | - | - |  | - |
| DC283 | E | - | - | - | - | - | - | - | - | - | - |  | - |
| *Pantoea* sp. |  |  |  |  |  |  |  |  |  |  |  |  |  |
| TX9 | C |  |  | - | - |  |  |  | - | - |  |  | - |

^*^C, clinical; E, environment; -/+, initial survey test was negative but subsequent testing was positive

^a^standard overlay assay spotted with 5 µL test bacteria

^b^standard overlay assay streaked with individual colony via toothpick

^c^portion of data reported previously (Walterson et al., 2014; Robinson et al., 2020; Williams and Stavrinides, 2020)

^d^tested with wild-type strain (3581 or SN01080)

Supplementary Table 4. Summary of antibiotic production by *Pantoea* species.*

| Strains tested | | Antibiotic producers | | Multi-strain activity | | G- and G+ | | G- activity | | G+ activity | |
| --- | --- | --- | --- | --- | --- | --- | --- | --- | --- | --- | --- |
| Species | No. | No. | % | No. | % | No. | % | No. | % | No. | % |
| *P. agglomerans* | 60 | 40 | 66.7 | 30 | 50.0 | 17 | 28.3 | 30 | 50.0 | 27 | 45.0 |
| *P. ananatis* | 10 | 3 | 30.0 | 1 | 10.0 | 0 | 0.0 | 3 | 30.0 | 0 | 0.0 |
| *P. anthophila* | 1 | 1 | 100.0 | 0 | 0.0 | 0 | 0.0 | 0 | 0.0 | 1 | 100.0 |
| *P. brenneri* | 5 | 2 | 40.0 | 1 | 20.0 | 0 | 0.0 | 1 | 20.0 | 1 | 20.0 |
| *P. conspicua* | 1 | 1 | 100.0 | 0 | 0.0 | 0 | 0.0 | 1 | 100.0 | 0 | 0.0 |
| *P. dispersa* | 3 | 3 | 100.0 | 2 | 66.7 | 1 | 33.3 | 2 | 66.7 | 2 | 66.7 |
| *P. eucalypti* | 8 | 3 | 37.5 | 2 | 25.0 | 1 | 12.5 | 1 | 12.5 | 3 | 37.5 |
| *P. eucrina* | 3 | 1 | 33.3 | 0 | 0.0 | 0 | 0.0 | 1 | 33.3 | 0 | 0.0 |
| *P. latae* | 2 | 0 | 0.0 | 0 | 0.0 | 0 | 0.0 | 0 | 0.0 | 0 | 0.0 |
| *P. septica* | 20 | 5 | 25.0 | 0 | 0.0 | 0 | 0.0 | 0 | 0.0 | 5 | 25.0 |
| *P. stewartii* | 2 | 0 | 0.0 | 0 | 0.0 | 0 | 0.0 | 0 | 0.0 | 0 | 0.0 |
| *Pantoea* sp. | 1 | 0 | 0.0 | 0 | 0.0 | 0 | 0.0 | 0 | 0.0 | 0 | 0.0 |
|  |  |  |  |  |  |  |  |  |  |  |  |
| Totals: | 116 | 59 | 50.9 | 36 | 31.0 | 19 | 16.4 | 39 | 33.6 | 39 | 33.6 |

*No., number; G-, Gram negative; G+, Gram positive; %, percentage of active strains per *Pantoea* species

Supplementary Table 5. Summary of antibiotic producing strains.*

| Strain | No. susceptible | % susceptible | No. GN susceptible | % GN susceptible | No. GP susceptible | % GP susceptible |
| --- | --- | --- | --- | --- | --- | --- |
| *P. agglomerans* |  |  |  |  |  |  |
| 22 | 1 | 100.0 | 1 | 100.0 | 0 |  |
| 23 | 1 | 100.0 | 1 | 100.0 | 0 |  |
| 83 | 0 | 0.0 | 0 | 0.0 | 0 | 0.0 |
| 788 | 0 | 0.0 | 0 | 0.0 | 0 | 0.0 |
| 1512 | 0 | 0.0 | 0 | 0.0 | 0 | 0.0 |
| 1574 | 0 | 0.0 | 0 | 0.0 | 0 | 0.0 |
| 3581r | 8 | 66.7 | 8 | 88.9 | 0 | 0.0 |
| 5565 | 0 | 0.0 | 0 | 0.0 | 0 | 0.0 |
| 7373 | 3 | 25.0 | 3 | 33.3 | 0 | 0.0 |
| 7612 | 3 | 25.0 | 3 | 33.3 | 0 | 0.0 |
| 12531 | 2 | 16.7 | 2 | 22.2 | 0 | 0.0 |
| 12534 | 0 | 0.0 | 0 | 0.0 | 0 | 0.0 |
| 13301 | 4 | 33.3 | 2 | 22.2 | 2 | 66.7 |
| 17124 | 0 | 0.0 | 0 | 0.0 | 0 | 0.0 |
| 770398 | 3 | 27.3 | 3 | 33.3 | 0 | 0.0 |
| 20S | 1 | 100.0 | 1 | 100.0 | 0 |  |
| 240R | 0 | 0.0 | 0 | 0.0 | 0 | 0.0 |
| 308R | 1 | 8.3 | 0 | 0.0 | 1 | 33.3 |
| B015092 | 0 | 0.0 | 0 | 0.0 | 0 | 0.0 |
| B016395 | 3 | 25.0 | 1 | 11.1 | 2 | 66.7 |
| B025670 | 6 | 60.0 | 6 | 66.7 | 0 | 0.0 |
| B026440 | 6 | 50.0 | 4 | 44.4 | 2 | 66.7 |
| B23I | 1 | 100.0 | 1 | 100.0 | 0 |  |
| BB834250 | 1 | 9.1 | 1 | 11.1 | 0 | 0.0 |
| DB522094 | 0 | 0.0 | 0 | 0.0 | 0 | 0.0 |
| DC432 | 0 | 0.0 | 0 | 0.0 | 0 | 0.0 |
| DC434 | 6 | 75.0 | 3 | 60.0 | 3 | 100.0 |
| DC556 | 0 | 0.0 | 0 | 0.0 | 0 | 0.0 |
| EH318 | 6 | 54.5 | 6 | 66.7 | 0 | 0.0 |
| G4032547 | 0 | 0.0 | 0 | 0.0 | 0 | 0.0 |
| H42501 | 0 | 0.0 | 0 | 0.0 | 0 | 0.0 |
| SN01080r | 11 | 91.7 | 9 | 100.0 | 2 | 66.7 |
| SN01121 | 2 | 28.6 | 0 | 0.0 | 2 | 100.0 |
| SN01122 | 2 | 16.7 | 0 | 0.0 | 2 | 66.7 |
| SN01170 | 2 | 16.7 | 1 | 11.1 | 1 | 33.3 |
| SP00101 | 0 | 0.0 | 0 | 0.0 | 0 | 0.0 |
| SP00202 | 2 | 28.6 | 0 | 0.0 | 2 | 100.0 |
| SP00303 | 1 | 14.3 | 0 | 0.0 | 1 | 50.0 |
| SP01202 | 7 | 63.6 | 6 | 66.7 | 1 | 50.0 |
| SP01220 | 1 | 14.3 | 0 | 0.0 | 1 | 50.0 |
| SP01230 | 0 | 0.0 | 0 | 0.0 | 0 | 0.0 |
| SP02022 | 2 | 18.2 | 0 | 0.0 | 2 | 66.7 |
| SP02230 | 2 | 18.2 | 0 | 0.0 | 2 | 66.7 |
| SP02243 | 3 | 25.0 | 1 | 11.1 | 2 | 66.7 |
| SP03310 | 0 | 0.0 | 0 | 0.0 | 0 | 0.0 |
| SP03383 | 2 | 18.2 | 1 | 12.5 | 1 | 33.3 |
| SP03412 | 6 | 54.5 | 5 | 55.6 | 1 | 50.0 |
| SP04010 | 3 | 42.9 | 1 | 20.0 | 2 | 100.0 |
| SP04011 | 1 | 9.1 | 0 | 0.0 | 1 | 33.3 |
| SP04021 | 4 | 33.3 | 2 | 22.2 | 2 | 66.7 |
| SP04022 | 3 | 27.3 | 1 | 12.5 | 2 | 66.7 |
| SP05051 | 0 | 0.0 | 0 | 0.0 | 0 | 0.0 |
| SP05052 | 3 | 27.3 | 1 | 12.5 | 2 | 66.7 |
| SP05061 | 4 | 57.1 | 2 | 40.0 | 2 | 100.0 |
| SP05092 | 1 | 8.3 | 0 | 0.0 | 1 | 33.3 |
| SP05120 | 8 | 66.7 | 5 | 55.6 | 3 | 100.0 |
| SP05130 | 0 | 0.0 | 0 | 0.0 | 0 | 0.0 |
| SS02010 | 0 | 0.0 | 0 | 0.0 | 0 | 0.0 |
| SS03231 | 2 | 33.3 | 2 | 40.0 | 0 | 0.0 |
| TX10 | 7 | 63.6 | 5 | 55.6 | 2 | 100.0 |
| *P. ananatis* |  |  |  |  |  |  |
| 15320 | 0 | 0.0 | 0 | 0.0 | 0 | 0.0 |
| 17671 | 0 | 0.0 | 0 | 0.0 | 0 | 0.0 |
| 26SR6 | 0 | 0.0 | 0 | 0.0 | 0 | 0.0 |
| B7 | 1 | 16.7 | 1 | 20.0 | 0 | 0.0 |
| BRT175 | 4 | 33.3 | 4 | 44.4 | 0 | 0.0 |
| BRT98 | 0 | 0.0 | 0 | 0.0 | 0 | 0.0 |
| Cit30-11R | 0 | 0.0 | 0 | 0.0 | 0 | 0.0 |
| LMG20103 | 0 | 0.0 | 0 | 0.0 | 0 | 0.0 |
| LMG5342 | 0 | 0.0 | 0 | 0.0 | 0 | 0.0 |
| M232A | 1 | 9.1 | 1 | 11.1 | 0 | 0.0 |
| *P. anthophila* |  |  |  |  |  |  |
| 1373 | 1 | 9.1 | 0 | 0.0 | 1 | 50.0 |
| *P. brenneri* |  |  |  |  |  |  |
| 91151 | 0 | 0.0 | 0 | 0.0 | 0 | 0.0 |
| B011483 | 1 | 10.0 | 1 | 12.5 | 0 | 0.0 |
| B014130 | 2 | 16.7 | 0 | 0.0 | 2 | 66.7 |
| B016381 | 0 | 0.0 | 0 | 0.0 | 0 | 0.0 |
| B024858 | 0 | 0.0 | 0 | 0.0 | 0 | 0.0 |
| *P. conspicua* |  |  |  |  |  |  |
| B011017 | 1 | 8.3 | 1 | 11.1 | 0 | 0.0 |
| *P. dispersa* |  |  |  |  |  |  |
| 625 | 1 | 9.1 | 0 | 0.0 | 1 | 50.0 |
| M1657A | 4 | 36.4 | 4 | 44.4 | 0 | 0.0 |
| M1657B | 6 | 54.5 | 5 | 55.6 | 1 | 50.0 |
| *P. eucalypti* |  |  |  |  |  |  |
| 299R | 0 | 0.0 | 0 | 0.0 | 0 | 0.0 |
| B011489 | 0 | 0.0 | 0 | 0.0 | 0 | 0.0 |
| F9026 | 0 | 0.0 | 0 | 0.0 | 0 | 0.0 |
| SM03214 | 1 | 20.0 | 0 | 0.0 | 1 | 100.0 |
| SP02021 | 3 | 42.9 | 1 | 20.0 | 2 | 100.0 |
| SP03372 | 0 | 0.0 | 0 | 0.0 | 0 | 0.0 |
| SP03391 | 3 | 25.0 | 0 | 0.0 | 3 | 100.0 |
| SP04013 | 0 | 0.0 | 0 | 0.0 | 0 | 0.0 |
| *P. eucrina* |  |  |  |  |  |  |
| 6868 | 0 | 0.0 | 0 | 0.0 | 0 | 0.0 |
| TX5 | 0 | 0.0 | 0 | 0.0 | 0 | 0.0 |
| TX6 | 1 | 9.1 | 1 | 11.1 | 0 | 0.0 |
| *P. latae* |  |  |  |  |  |  |
| TX11 | 0 | 0.0 | 0 | 0.0 | 0 | 0.0 |
| TX7 | 0 | 0.0 | 0 | 0.0 | 0 | 0.0 |
| *P. septica* |  |  |  |  |  |  |
| 101150 | 0 | 0.0 | 0 | 0.0 | 0 | 0.0 |
| 062465A | 1 | 9.1 | 0 | 0.0 | 1 | 50.0 |
| 062465B | 0 | 0.0 | 0 | 0.0 | 0 | 0.0 |
| 091957A | 1 | 9.1 | 0 | 0.0 | 1 | 50.0 |
| 091957B | 0 | 0.0 | 0 | 0.0 | 0 | 0.0 |
| 81828 | 0 | 0.0 | 0 | 0.0 | 0 | 0.0 |
| B016375 | 0 | 0.0 | 0 | 0.0 | 0 | 0.0 |
| BB350028A | 1 | 9.1 | 0 | 0.0 | 1 | 50.0 |
| BB350028B | 1 | 9.1 | 0 | 0.0 | 1 | 50.0 |
| BE528629 | 0 | 0.0 | 0 | 0.0 | 0 | 0.0 |
| G2291404 | 0 | 0.0 | 0 | 0.0 | 0 | 0.0 |
| G3271436 | 1 | 9.1 | 0 | 0.0 | 1 | 50.0 |
| G4071105 | 0 | 0.0 | 0 | 0.0 | 0 | 0.0 |
| M1517 | 0 | 0.0 | 0 | 0.0 | 0 | 0.0 |
| M41864 | 0 | 0.0 | 0 | 0.0 | 0 | 0.0 |
| TX3 | 0 | 0.0 | 0 | 0.0 | 0 | 0.0 |
| TX4 | 0 | 0.0 | 0 | 0.0 | 0 | 0.0 |
| VB38951A | 0 | 0.0 | 0 | 0.0 | 0 | 0.0 |
| VB38951B | 0 | 0.0 | 0 | 0.0 | 0 | 0.0 |
| X44686 | 0 | 0.0 | 0 | 0.0 | 0 | 0.0 |
| *P. stewartii* |  |  |  |  |  |  |
| 626 | 0 | 0.0 | 0 | 0.0 | 0 | 0.0 |
| DC283 | 0 | 0.0 | 0 | 0.0 | 0 | 0.0 |
| *Pantoea* sp. |  |  |  |  |  |  |
| TX9 | 0 | 0.0 | 0 | 0.0 | 0 | 0.0 |

*No., number; G-, Gram negative; G+, Gram positive; %, percentage of pathogens tested that were susceptible to individual *Pantoea* strains

Supplementary Table 6. antiSMASH analysis of *Pantoea* genomes.

| **Strain** | **Region** | **Type** | **Most similar known cluster** | **Type** | **Similarity** | **Contig end?** |
| --- | --- | --- | --- | --- | --- | --- |
| *P. agglomerans* |  |  |  |  |  |  |
| 3581 | 1.1 | Saccharide |  |  |  | N |
| (25 hits) | 1.2 | Saccharide |  |  |  | N |
|  | 1.3 | Saccharide | Polysaccharide B | Saccharide | 6% | N |
|  | 1.4 | Saccharide | Surfactin | NRP:Lipopeptide | 8% | N |
|  | 1.5 | Saccharide | Stewartan | Saccharide | 92% | N |
|  | 1.6 | Fatty acid |  |  |  | N |
|  | 2.1 | NRPS* | Turnerbactin | NRP | 30% | N |
|  | 2.2 | Hserlactone* |  |  |  | N |
|  | 2.3 | Saccharide |  |  |  | N |
|  | 3.1 | Siderophore* | Desferrioxamine E | Other | 100% | N |
|  | 3.2 | Saccharide |  |  |  | N |
|  | 3.3 | Terpene* | Carotenoid | Terpene | 100% | N |
|  | 5.1 | Saccharide |  |  |  | N |
|  | 5.2 | Fatty acid |  |  |  | N |
|  | 5.3 | Arylpolyene*  Saccharide  Hserlactone* | Aryl polyenes | Other | 94% | N |
|  | 5.4 | Saccharide | Taxlllaid A | NRP | 13% | N |
|  | 5.5 | Thiopeptide* | O-antigen | Saccharide | 14% | N |
|  | 7.1 | Saccharide |  |  |  | N |
|  | 7.2 | Saccharide | Emulsan | Saccharide | 9% | N |
|  | 8.1 | Saccharide |  |  |  | N |
|  | 9.1 | Saccharide |  |  |  | N |
|  | 10.1 | Saccharide |  |  |  | N |
|  | 10.2 | Fatty acid | Herboxidiene | Polyketide | 2% | N |
|  | 11.1 | Saccharide | O&K-antigen | Saccharide | 4% | N |
|  | 13.1 | Phosphonate*  NRPS* |  |  |  | Y |
|  |  |  |  |  |  |  |
| B025670 | 1.1 | Fatty acid |  |  |  | N |
| (24 hits) | 1.2 | Arylpolyene*  Saccharide  Hserlactone* | Aryl polyenes | Other | 94% | N |
|  | 1.3 | Saccharide | Taxlllaid A | NRP | 13% | N |
|  | 1.4 | Thiopeptide* | O-antigen | Saccharide | 14% | N |
|  | 2.1 | Saccharide |  |  |  | N |
|  | 4.1 | Fatty acid |  |  |  | N |
|  | 6.1 | Terpene* | Carotenoid | Terpene | 100% | N |
|  | 8.1 | Saccharide |  |  |  | N |
|  | 10.1 | Saccharide |  |  |  | N |
|  | 15.1 | Saccharide | Emulsan | Saccharide | 9% | N |
|  | 15.2 | Saccharide |  |  |  | N |
|  | 18.1 | Saccharide | Stewartan | Saccharide | 92% | N |
|  | 18.2 | Fatty acid |  |  |  | N |
|  | 21.1 | Siderophore* | Desferrioxamine E | Other | 100% | N |
|  | 21.2 | Saccharide |  |  |  | N |
|  | 21.3 | Saccharide |  |  |  | N |
|  | 23.1 | Saccharide | O&K-antigen | Saccharide | 4% | N |
|  | 30.1 | Saccharide | Polysaccharide B | Saccharide | 6% | N |
|  | 30.2 | Saccharide |  |  |  | N |
|  | 31.1 | Saccharide |  |  |  | N |
|  | 31.2 | Fatty acid | Herboxidiene | Polyketide | 2% | N |
|  | 32.1 | Saccharide |  |  |  | N |
|  | 32.2 | Hserlactone* |  |  |  | N |
|  | 32.3 | NRPS* | Amonabactin P 750 | NRP | 57% | N |
|  |  |  |  |  |  |  |
| DC432 | 1.1 | Fatty acid |  |  |  | N |
| (24 hits) | 6.1 | Saccharide | O&K-antigen | Saccharide | 4% | N |
|  | 9.1 | Fatty acid | Herboxidiene | Polyketide | 2% | N |
|  | 9.2 | Saccharide |  |  |  | N |
|  | 14.1 | Terpene* | Carotenoid | Terpene | 100% | N |
|  | 14.2 | Saccharide |  |  |  | N |
|  | 14.3 | Siderophore* | Desferrioxamine E | Other | 100% | N |
|  | 15.1 | Saccharide |  |  |  | N |
|  | 15.2 | Saccharide | Emulsan | Saccharide | 9% | N |
|  | 16.1 | Saccharide | Stewartan | Saccharide | 92% | N |
|  | 22.1 | Saccharide |  |  |  | N |
|  | 25.1 | Saccharide | Polysaccharide B | Saccharide | 6% | N |
|  | 25.2 | Saccharide |  |  |  | N |
|  | 25.3 | Saccharide |  |  |  | N |
|  | 26.1 | Saccharide |  |  |  | N |
|  | 26.2 | Fatty acid |  |  |  | N |
|  | 26.3 | Arylpolyene*  Saccharide | Aryl polyenes | Other | 94% | N |
|  | 26.4 | Hserlactone* |  |  |  | N |
|  | 26.5 | Saccharide | Taxlllaid A | NRP | 13% | N |
|  | 26.6 | Thiopeptide* | O-antigen | Saccharide | 14% | N |
|  | 27.1 | Saccharide |  |  |  | N |
|  | 27.2 | Hserlactone* |  |  |  | N |
|  | 27.3 | NRPS* | Amonabactin P 750 | NRP | 57% | N |
|  | 28.1 | Saccharide |  |  |  | N |
|  |  |  |  |  |  |  |
| EH318 | 2.1 | Fatty acid |  |  |  | N |
| (22 hits) | 4.1 | Saccharide | Polysaccharide B | Saccharide | 6% | N |
|  | 7.1 | Saccharide |  |  |  | N |
|  | 9.1 | Saccharide |  |  |  | Y |
|  | 9.2 | Siderophore* | Desferrioxamine E | Other | 100% | N |
|  | 9.3 | Terpene* | Carotenoid | Terpene | 100% | N |
|  | 14.1 | Saccharide | O&K-antigen | Saccharide | 4% | N |
|  | 22.1 | Saccharide | Emulsan | Saccharide | 9% | N |
|  | 22.2 | Saccharide |  |  |  | N |
|  | 26.1 | Saccharide |  |  |  | N |
|  | 29.1 | Hserlactone* |  |  |  | N |
|  | 29.2 | NRPS* | Amonabactin P 750 | NRP | 57% | N |
|  | 30.1 | Saccharide | Stewartan | Saccharide | 92% | N |
|  | 31.1 | Thiopeptide* | O-antigen | Saccharide | 14% | N |
|  | 31.2 | Saccharide | Taxlllaid A | NRP | 13% | N |
|  | 31.3 | Hserlactone*  Arylpolyene*  Saccharide | Aryl polyenes | Other | 94% | N |
|  | 31.4 | Fatty acid |  |  |  | N |
|  | 31.5 | Saccharide |  |  |  | N |
|  | 31.6 | Saccharide |  |  |  | N |
|  | 32.1 | Saccharide |  |  |  | N |
|  | 32.2 | Fatty acid | Herboxidiene | Polyketide | 2% | N |
|  | 33.1 | Saccharide |  |  |  | N |
|  |  |  |  |  |  |  |
| SN01080 | 1.1 | Thiopeptide* | O-antigen | Saccharide | 14% | N |
| (24 hits) | 1.2 | Saccharide | Taxlllaid A | NRP | 13% | N |
|  | 1.3 | Hserlactone*  Arylpolyene*  Saccharide | Aryl polyenes | Other | 94% | N |
|  | 1.4 | Fatty acid |  |  |  | N |
|  | 1.5 | Saccharide |  |  |  | N |
|  | 1.6 | Saccharide |  |  |  | N |
|  | 1.7 | Saccharide |  |  |  | N |
|  | 1.8 | Saccharide | Polysaccharide B | Saccharide | 6% | N |
|  | 2.1 | Saccharide |  |  |  | N |
|  | 2.2 | Saccharide | Stewartan | Saccharide | 92% | N |
|  | 2.3 | Fatty acid |  |  |  | N |
|  | 2.4 | NRPS* |  |  |  | N |
|  | 3.1 | Saccharide |  |  |  | N |
|  | 3.2 | Hserlactone* |  |  |  | N |
|  | 3.3 | NRPS* | Amonabactin P 750 | NRP | 57% | N |
|  | 4.1 | Terpene* | Carotenoid | Terpene | 100% | N |
|  | 4.2 | Siderophore* | Desferrioxamine E | Other | 100% | N |
|  | 4.3 | Saccharide |  |  |  | N |
|  | 4.4 | Saccharide |  |  |  | N |
|  | 5.1 | Saccharide |  |  |  | N |
|  | 7.1 | Saccharide | Emulsan | Saccharide | 9% | N |
|  | 8.1 | Fatty acid |  |  |  | Y |
|  | 8.2 | Saccharide |  |  |  | N |
|  | 9.1 | Saccharide | O&K-antigen | Saccharide | 4% | N |
|  |  |  |  |  |  |  |
| SP00101 | 1.1 | Siderophore* | Desferrioxamine E | Other | 100% | Y |
| (28 hits) | 1.2 | Saccharide |  |  |  | N |
|  | 2.1 | Terpene* | Carotenoid | Terpene | 100% | N |
|  | 3.1 | Lassopeptide* |  |  |  | Y |
|  | 4.1 | Lassopeptide* |  |  |  | Y |
|  | 8.1 | NRPS* | Amonabactin P 750 | NRP | 57% | N |
|  | 8.2 | Hserlactone* |  |  |  | N |
|  | 8.3 | Saccharide |  |  |  | N |
|  | 9.1 | Saccharide | Lipopolysaccharide | Saccharide | 27% | N |
|  | 13.1 | Saccharide |  |  |  | N |
|  | 14.1 | Fatty acid |  |  |  | N |
|  | 14.2 | Saccharide |  |  |  | N |
|  | 14.3 | Fatty acid |  |  |  | N |
|  | 14.4 | Saccharide |  |  |  | N |
|  | 18.1 | Saccharide |  |  |  | N |
|  | 18.2 | Fatty acid |  |  |  | N |
|  | 18.3 | Arylpolyene*  Saccharide | Aryl polyenes | Other | 94% | N |
|  | 18.4 | Hserlactone* |  |  |  | N |
|  | 18.5 | Saccharide | Taxlllaid A | NRP | 13% | N |
|  | 18.6 | Thiopeptide* | O-antigen | Saccharide | 14% | N |
|  | 20.1 | Saccharide |  |  |  | N |
|  | 24.1 | Saccharide | Stewartan | Saccharide | 92% | N |
|  | 24.2 | Fatty acid |  |  |  | N |
|  | 25.1 | Saccharide |  |  |  | N |
|  | 25.2 | Saccharide | Emulsan | Saccharide | 9% | N |
|  | 26.1 | Saccharide |  |  |  | N |
|  | 27.1 | Saccharide |  |  |  | N |
|  | 27.2 | Saccharide | Polysaccharide B | Saccharide | 6% | N |
|  |  |  |  |  |  |  |
| SP04022 | 1.1 | Fatty acid |  |  |  | N |
| (22 hits) | 1.2 | Saccharide |  |  |  | N |
|  | 4.1 | Saccharide | O&K-antigen | Saccharide | 4% | N |
|  | 7.1 | Saccharide |  |  |  | Y |
|  | 7.2 | Siderophore* | Desferrioxamine E | Other | 100% | N |
|  | 7.3 | Terpene* | Carotenoid | Terpene | 100% | N |
|  | 8.1 | Saccharide |  |  |  | N |
|  | 10.1 | Fatty acid |  |  |  | N |
|  | 10.2 | Saccharide |  |  |  | N |
|  | 12.1 | Fatty acid |  |  |  | N |
|  | 17.1 | Saccharide | O&K-antigen | Saccharide | 3% | N |
|  | 19.1 | Thiopeptide* | O-antigen | Saccharide | 14% | N |
|  | 19.2 | Saccharide | Taxlllaid A | NRP | 13% | N |
|  | 19.3 | Hserlactone*  Arylpolyene*  Saccharide | Aryl polyenes | Other | 94% | Y |
|  | 21.1 | Saccharide |  |  |  | N |
|  | 23.1 | NRPS* | Amonabactin P 750 | NRP | 57% | N |
|  | 23.2 | Hserlactone* | Colicin V | RiPP | 1% | N |
|  | 23.3 | Saccharide |  |  |  | N |
|  | 24.1 | Saccharide |  |  |  | N |
|  | 24.2 | Saccharide | Polysaccharide B | Saccharide | 6% | N |
|  | 24.3 | Saccharide |  |  |  | N |
|  | 24.4 | Saccharide | Stewartan | Saccharide | 92% | N |
|  |  |  |  |  |  |  |
| TX10 | 1.1 | Saccharide |  |  |  | N |
| (23 hits) | 2.1 | Saccharide |  |  |  | N |
|  | 4.1 | Arylpolyene*  Saccharide | Aryl polyenes | Other | 94% | Y |
|  | 13.1 | Saccharide | Stewartan | Saccharide | 92% | N |
|  | 13.2 | Fatty acid |  |  |  | N |
|  | 14.1 | Saccharide |  |  |  | N |
|  | 15.1 | Saccharide |  |  |  | N |
|  | 15.2 | Saccharide | O&K-antigen | Saccharide | 3% | N |
|  | 17.1 | Hserlactone* |  |  |  | Y |
|  | 17.2 | Saccharide | Taxlllaid A | NRP | 13% | N |
|  | 17.3 | Thiopeptide* | O-antigen | Saccharide | 14% | N |
|  | 21.1 | Siderophore* | Desferrioxamine E | Other | 100% | N |
|  | 21.2 | Terpene* | Carotenoid | Terpene | 100% | N |
|  | 23.1 | Saccharide |  |  |  | N |
|  | 23.2 | Fatty acid | Herboxidiene | Polyketide | 2% | N |
|  | 24.1 | Fatty acid |  |  |  | Y |
|  | 24.2 | Saccharide |  |  |  | N |
|  | 24.3 | Saccharide |  |  |  | N |
|  | 24.4 | Saccharide | Polysaccharide B | Saccharide | 6% | N |
|  | 25.1 | Saccharide | O&K-antigen | Saccharide | 4% | N |
|  | 26.1 | Saccharide |  |  |  | N |
|  | 26.2 | Hserlactone* |  |  |  | N |
|  | 26.3 | NRPS* | Amonabactin P 750 | NRP | 57% | N |
|  |  |  |  |  |  |  |
| *P. ananatis* |  |  |  |  |  |  |
| 15320 | 1.1 | Terpene* | Carotenoid | Terpene | 100% | N |
| (21 hits) | 2.1 | Saccharide |  |  |  | N |
|  | 25.1 | Siderophore* | Aerobactin | Other | 77% | N |
|  | 25.2 | Saccharide |  |  |  | N |
|  | 26.1 | Saccharide |  |  |  | N |
|  | 39.1 | Saccharide |  |  |  | N |
|  | 43.1 | Saccharide | Emulsan | Saccharide | 9% | N |
|  | 44.1 | Saccharide | O&K-antigen | Saccharide | 4% | N |
|  | 45.1 | Hserlactone*  Saccharide |  |  |  | N |
|  | 45.2 | Saccharide | Polysaccharide B | Saccharide | 6% | N |
|  | 45.3 | Saccharide | Lipopolysaccharide | Saccharide | 18% | Y |
|  | 47.1 | Saccharide | Stewartan | Saccharide | 92% | N |
|  | 47.2 | Saccharide | Entolysin | NRP | 8% | N |
|  | 47.3 | Fatty acid |  |  |  | N |
|  | 48.1 | Arylpolyene*  Saccharide | APE Ec | Other | 94% | N |
|  | 48.2 | Siderophore* | Desferrioxamine E | Other | 100% | N |
|  | 48.3 | Saccharide |  |  |  | N |
|  | 49.1 | Thiopeptide* | O-antigen | Saccharide | 14% | N |
|  | 49.2 | Saccharide | Taxlllaid A | NRP | 13% | N |
|  | 49.3 | Hserlactone* |  |  |  | N |
|  | 49.4 | Fatty acid |  |  |  | N |
|  |  |  |  |  |  |  |
| 17671 | 1.1 | Fatty acid |  |  |  | N |
| (20 hits) | 1.2 | Saccharide | Stewartan | Saccharide | 92% | N |
|  | 1.3 | Saccharide | Lipopolysaccharide | Saccharide | 18% | N |
|  | 1.4 | Saccharide | Polysaccharide B | Saccharide | 6% | N |
|  | 2.1 | Hserlactone* |  |  |  | N |
|  | 2.2 | Saccharide | Taxlllaid A | NRP | 13% | N |
|  | 2.3 | Thiopeptide* | O-antigen | Saccharide | 14% | N |
|  | 3.1 | Siderophore* | Aerobactin | Other | 77% | N |
|  | 3.2 | Saccharide |  |  |  | N |
|  | 4.1 | Fatty acid |  |  |  | N |
|  | 4.2 | Siderophore* | Desferrioxamine E | Other | 100% | N |
|  | 4.3 | Saccharide |  |  |  | N |
|  | 6.1 | Saccharide  Hserlactone* |  |  |  | N |
|  | 8.1 | Saccharide |  |  |  | N |
|  | 9.1 | Saccharide |  |  |  | N |
|  | 11.1 | Saccharide | Emulsan | Saccharide | 9% | Y |
|  | 12.1 | Fatty acid |  |  |  | N |
|  | 13.1 | Saccharide |  |  |  | N |
|  | 14.1 | Saccharide | O&K-antigen | Saccharide | 4% | N |
|  | 18.1 | Terpene* | Carotenoid | Terpene | 100% | N |
|  |  |  |  |  |  |  |
| 26SR6  (21 hits) | 1.1 | Hserlactone*  Saccharide |  |  |  | N |
|  | 1.2 | Saccharide | Polysaccharide B | Saccharide | 6% | N |
|  | 1.3 | Saccharide | N-myristoyl-D-asparagine  cis-7-tetradecenoyl-D-asparagine  (R)-N1-((S)-5-oxohexan-2-yl)-2-tetradecanamidosuccinamide | NRP  Polyketide:Modular type I  Polyketide:Trans-AT type I | 8% | N |
|  | 1.4 | Saccharide | Stewartan | Saccharide | 92% | N |
|  | 1.5 | Saccharide | Entolysin | NRP | 8% | N |
|  | 1.6 | Fatty acid |  |  |  | N |
|  | 2.1 | Thiopeptide* | O-antigen | Saccharide | 14% | N |
|  | 2.2 | Saccharide | Taxlllaid A | NRP | 13% | N |
|  | 2.3 | Hserlactone* |  |  |  | N |
|  | 2.4 | Fatty acid |  |  |  | N |
|  | 3.1 | Saccharide |  |  |  | N |
|  | 3.2 | Siderophore* | Desferrioxamine E | Other | 100% | N |
|  | 3.3 | Saccharide |  |  |  | N |
|  | 3.4 | Siderophore* | Aerobactin | Other | 77% | N |
|  | 5.1 | Saccharide |  |  |  | N |
|  | 6.1 | Terpene* | Carotenoid | Terpene | 100% | N |
|  | 6.2 | Saccharide |  |  |  | N |
|  | 7.1 | Saccharide | Emulsan | Saccharide | 9% | N |
|  | 8.1 | Saccharide |  |  |  | N |
|  | 9.1 | Saccharide |  |  |  | N |
|  | 10.1 | Saccharide | O&K-antigen | Saccharide | 4% | N |
|  |  |  |  |  |  |  |
| B7 | 1.1 | Saccharide |  |  |  | N |
| (21 hits) | 1.2 | Hserlactone*  Saccharide |  |  |  | N |
|  | 1.3 | Saccharide | Polysaccharide B | Saccharide | 6% | N |
|  | 1.4 | Saccharide | Lipopolysaccharide | Saccharide | 18% | Y |
|  | 2.1 | Saccharide |  |  |  | N |
|  | 2.2 | Siderophore* | Desferrioxamine E | Other | 100% | N |
|  | 2.3 | Arylpolyene*  Saccharide | APE Ec | Other | 94% | N |
|  | 3.1 | Fatty acid |  |  |  | N |
|  | 3.2 | Hserlactone* |  |  |  | N |
|  | 3.3 | Saccharide | Taxlllaid A | NRP | 13% | N |
|  | 3.4 | Thiopeptide* | O-antigen | Saccharide | 14% | N |
|  | 5.1 | Saccharide | Entolysin | NRP | 8% | N |
|  | 5.2 | Fatty acid |  |  |  | N |
|  | 6.1 | Saccharide | Stewartan | Saccharide | 92% | N |
|  | 8.1 | Saccharide | Emulsan | Saccharide | 9% | N |
|  | 9.1 | Saccharide |  |  |  | N |
|  | 10.1 | Saccharide |  |  |  | N |
|  | 10.2 | Terpene* | Carotenoid | Terpene | 100% | N |
|  | 11.1 | Saccharide |  |  |  | Y |
|  | 11.2 | Siderophore* | Aerobactin | Other | 77% | N |
|  | 12.1 | Saccharide | O&K-antigen | Saccharide | 4% | N |
|  |  |  |  |  |  |  |
| BRT175 | 4.1 | Saccharide |  |  |  | Y |
| (20 hits) | 10.1 | Saccharide |  |  |  | N |
|  | 10.2 | Siderophore* | Aerobactin | Other | 77% | N |
|  | 12.1 | Hserlactone*  Saccharide |  |  |  | N |
|  | 15.1 | Saccharide | Stewartan | Saccharide | 92% | N |
|  | 18.1 | Saccharide | O&K-antigen | Saccharide | 4% | N |
|  | 19.1 | Saccharide |  |  |  | N |
|  | 19.2 | Siderophore* | Desferrioxamine E | Other | 100% | N |
|  | 19.3 | Arylpolyene*  Saccharide | APE Ec | Other | 94% | N |
|  | 23.1 | Fatty acid |  |  |  | N |
|  | 34.1 | Saccharide |  |  |  | N |
|  | 34.2 | Terpene* | Carotenoid | Terpene | 100% | N |
|  | 36.1 | Thiopeptide* | O-antigen | Saccharide | 14% | N |
|  | 36.2 | Saccharide | Taxlllaid A | NRP | 13% | N |
|  | 36.3 | Hserlactone* |  |  |  | Y |
|  | 39.1 | Saccharide |  |  |  | N |
|  | 40.1 | Saccharide | Polysaccharide B | Saccharide | 6% | N |
|  | 40.2 | Saccharide | Lipopolysaccharide | Saccharide | 18% | N |
|  | 41.1 | Fatty acid |  |  |  | N |
|  | 43.1 | Saccharide | Emulsan | Saccharide | 9% | N |
|  |  |  |  |  |  |  |
| BRT98 | 11.1 | Saccharide | Emulsan | Saccharide | 9% | N |
| (21 hits) | 12.1 | Terpene* | Carotenoid | Terpene | 100% | N |
|  | 12.2 | Saccharide |  |  |  | N |
|  | 12.3 | Terpene* | Carotenoid | Terpene | 100% | N |
|  | 14.1 | Saccharide | O&K-antigen | Saccharide | 4% | N |
|  | 17.1 | Saccharide |  |  |  | N |
|  | 20.1 | Fatty acid |  |  |  | N |
|  | 23.1 | Saccharide |  |  |  | N |
|  | 26.1 | Saccharide | Stewartan | Saccharide | 92% | N |
|  | 26.2 | Saccharide | Entolysin | NRP | 8% | N |
|  | 26.3 | Fatty acid |  |  |  | N |
|  | 27.1 | Saccharide |  |  |  | N |
|  | 27.2 | Siderophore* | Desferrioxamine E | Other | 100% | N |
|  | 27.3 | Saccharide |  |  |  | N |
|  | 27.4 | Siderophore* | Aerobactin | Other | 77% | N |
|  | 28.1 | Saccharide | N-myristoyl-D-asparagine  cis-7-tetradecenoyl-D-asparagine  (R)-N1-((S)-5-oxohexan-2-yl)-2-tetradecanamidosuccinamide | NRP  Polyketide:Modular type I  Polyketide:Trans-AT type I | 8% | N |
|  | 28.2 | Saccharide | Polysaccharide B | Saccharide | 6% | N |
|  | 28.3 | Saccharide  Hserlactone* |  |  |  | N |
|  | 29.1 | Thiopeptide* | O-antigen | Saccharide | 14% | N |
|  | 29.2 | Saccharide | Taxlllaid A | NRP | 13% | N |
|  | 29.3 | Hserlactone* |  |  |  | Y |
|  |  |  |  |  |  |  |
| Cit30-11R | 1.1 | Saccharide |  |  |  | N |
| (22 hits) | 1.2 | Fatty acid |  |  |  | N |
|  | 1.3 | Hserlactone* |  |  |  | N |
|  | 1.4 | Saccharide | Taxlllaid A | NRP | 13% | N |
|  | 1.5 | Thiopeptide* | O-antigen | Saccharide | 14% | N |
|  | 2.1 | Siderophore* | Aerobactin | Other | 77% | N |
|  | 2.2 | Saccharide |  |  |  | N |
|  | 2.3 | Arylpolyene*  Saccharide | APE Ec | Other | 94% | N |
|  | 2.4 | Siderophore* | Desferrioxamine E | Other | 100% | N |
|  | 2.5 | Saccharide |  |  |  | N |
|  | 4.1 | Saccharide | Entolysin | NRP | 8% | N |
|  | 4.2 | Fatty acid |  |  |  | N |
|  | 5.1 | Hserlactone*  Saccharide |  |  |  | N |
|  | 5.2 | Saccharide | Polysaccharide B | Saccharide | 6% | N |
|  | 5.3 | Saccharide | Lipopolysaccharide | Saccharide | 18% | Y |
|  | 6.1 | Saccharide |  |  |  | N |
|  | 6.2 | Terpene* | Carotenoid | Terpene | 100% | N |
|  | 6.3 | NRPS*  T1PKS* | Lipopolysaccharide | Saccharide | 16% | Y |
|  | 7.1 | Saccharide | Stewartan | Saccharide | 92% | N |
|  | 8.1 | Saccharide | Emulsan | Saccharide | 9% | N |
|  | 9.1 | Saccharide |  |  |  | N |
|  | 10.1 | Saccharide | O&K-antigen | Saccharide | 4% | N |
|  |  |  |  |  |  |  |
| M232A | 1.1 | Saccharide |  |  |  | N |
| (21 hits) | 1.2 | Hserlactone*  Saccharide |  |  |  | N |
|  | 1.3 | Saccharide | Polysaccharide B | Saccharide | 6% | N |
|  | 1.4 | Saccharide | Lipopolysaccharide | Saccharide | 18% | N |
|  | 2.1 | Fatty acid |  |  |  | N |
|  | 2.2 | Hserlactone* |  |  |  | N |
|  | 2.3 | Saccharide | Taxlllaid A | NRP | 13% | N |
|  | 2.4 | Thiopeptide* | O-antigen | Saccharide | 14% | N |
|  | 3.1 | Saccharide |  |  |  | N |
|  | 3.2 | Siderophore* | Desferrioxamine E | Other | 100% | N |
|  | 3.3 | Arylpolyene*  Saccharide | APE Ec | Other | 94% | N |
|  | 5.1 | Saccharide | Entolysin | NRP | 8% | N |
|  | 5.2 | Fatty acid |  |  |  | N |
|  | 6.1 | Saccharide | Stewartan | Saccharide | 92% | N |
|  | 7.1 | Saccharide | Emulsan | Saccharide | 9% | N |
|  | 8.1 | Saccharide |  |  |  | N |
|  | 9.1 | Saccharide |  |  |  | N |
|  | 9.2 | Terpene* | Carotenoid | Terpene | 100% | N |
|  | 10.1 | Saccharide |  |  |  | Y |
|  | 10.2 | Siderophore* | Aerobactin | Other | 77% | N |
|  | 11.1 | Saccharide | O&K-antigen | Saccharide | 4% | N |
|  |  |  |  |  |  |  |
| *P. brenneri* |  |  |  |  |  |  |
| B016381 | 2.1 | Fatty acid |  |  |  | Y |
| (20 hits) | 4.1 | Saccharide | O-antigen | Saccharide | 10% | N |
|  | 4.2 | Saccharide |  |  |  | N |
|  | 5.1 | Saccharide | O&K-antigen | Saccharide | 4% | N |
|  | 9.1 | Terpene* | Carotenoid | Terpene | 100% | N |
|  | 15.1 | Fatty acid |  |  |  | N |
|  | 15.2 | Saccharide  Arylpolyene* | Aryl polyenes | Other | 94% | N |
|  | 15.3 | Saccharide | Taxlllaid A | NRP | 13% | N |
|  | 15.4 | Thiopeptide* | O-antigen | Saccharide | 14% | N |
|  | 16.1 | Fatty acid  Butyrolactone* |  |  |  | Y |
|  | 17.1 | Saccharide |  |  |  | Y |
|  | 20.1 | Fatty acid | Herboxidiene | Polyketide | 2% | Y |
|  | 24.1 | Saccharide | Stewartan | Saccharide | 92% | N |
|  | 30.1 | Saccharide |  |  |  | Y |
|  | 40.1 | Saccharide |  |  |  | N |
|  | 44.1 | NRPS* | Amonabactin P 750 | NRP | 57% | N |
|  | 54.1 | Saccharide |  |  |  | N |
|  | 56.1 | Saccharide | Emulsan | Saccharide | 9% | N |
|  | 58.1 | Saccharide | Polysaccharide B | Saccharide | 6% | N |
|  | 58.2 | Saccharide |  |  |  | N |
|  |  |  |  |  |  |  |
| B024858 | 5.1 | Saccharide | Emulsan | Saccharide | 9% | N |
| (23 hits) | 6.1 | Saccharide |  |  |  | N |
|  | 13.1 | Saccharide |  |  |  | N |
|  | 14.1 | Saccharide | Stewartan | Saccharide | 92% | N |
|  | 15.1 | Saccharide | Formicamycins A-M | Polyketide | 4% | N |
|  | 15.2 | Fatty acid |  |  |  | N |
|  | 16.1 | Terpene* | Carotenoid | Terpene | 100% | N |
|  | 22.1 | Saccharide |  |  |  | Y |
|  | 23.1 | Fatty acid | Herboxidiene | Polyketide | 2% | N |
|  | 24.1 | Saccharide |  |  |  | N |
|  | 24.2 | Fatty acid |  |  |  | N |
|  | 24.3 | Saccharide  Arylpolyene* | Aryl polyenes | Other | 94% | N |
|  | 24.4 | Saccharide | Taxlllaid A | NRP | 13% | N |
|  | 24.5 | Thiopeptide* | O-antigen | Saccharide | 14% | N |
|  | 27.1 | Saccharide |  |  |  | Y |
|  | 34.1 | Saccharide |  |  |  | N |
|  | 35.1 | Saccharide | Emulsan | Saccharide | 9% | N |
|  | 36.1 | Saccharide | O&K-antigen | Saccharide | 4% | N |
|  | 37.1 | Lassopeptide* |  |  |  | Y |
|  | 41.1 | NRPS* | Amonabactin P 750 | NRP | 57% | N |
|  | 41.2 | Saccharide |  |  |  | N |
|  | 45.1 | Saccharide | Polysaccharide B | Saccharide | 6% | N |
|  | 45.2 | Saccharide |  |  |  | N |
|  |  |  |  |  |  |  |
| *P. dispersa* |  |  |  |  |  |  |
| 625 | 5.1 | Fatty acid |  |  |  | Y |
| (21 hits) | 7.1 | Fatty acid |  |  |  | N |
|  | 7.2 | Hserlactone* |  |  |  | N |
|  | 7.3 | Saccharide | Taxlllaid A | NRP | 15% | N |
|  | 7.4 | Thiopeptide* | O-antigen | Saccharide | 14% | N |
|  | 8.1 | Saccharide |  |  |  | N |
|  | 8.2 | Terpene*  Saccharide | Carotenoid | Terpene | 100% | N |
|  | 8.3 | Saccharide  Acyl amino acids* | Pyoverdin | NRP | 1% | N |
|  | 9.1 | Saccharide |  |  |  | N |
|  | 18.1 | Saccharide |  |  |  | N |
|  | 18.2 | NRPS* | Amonabactin P 750 | NRP | 57% | N |
|  | 18.3 | Saccharide |  |  |  | N |
|  | 18.4 | Saccharide |  |  |  | N |
|  | 21.1 | Saccharide | Lipopolysaccharide | Saccharide | 80% | N |
|  | 25.1 | Saccharide | Lipopolysaccharide | Saccharide | 18% | Y |
|  | 26.1 | Saccharide |  |  |  | N |
|  | 32.1 | Saccharide | Stewartan | Saccharide | 85% | N |
|  | 32.2 | Saccharide | Yersiniabactin | NRP+Polyketide | 4% | N |
|  | 33.1 | Saccharide | Polysaccharide B | Saccharide | 6% | N |
|  | 34.1 | Saccharide |  |  |  | N |
|  | 35.1 | Saccharide | Emulsan | Saccharide | 9% | Y |
|  |  |  |  |  |  |  |
| M1657A | 5.1 | Arylpolyene* | APE Ec | Other | 31% | Y |
| (26 hits) | 5.2 | Fatty acid |  |  |  | N |
|  | 8.1 | Saccharide |  |  |  | Y |
|  | 23.1 | Saccharide | O-antigen | Saccharide | 10% | Y |
|  | 28.1 | Saccharide |  |  |  | N |
|  | 29.1 | Saccharide | Lipopolysaccharide | Saccharide | 31% | N |
|  | 29.2 | Saccharide | Yersiniabactin | NRP+Polyketide | 4% | N |
|  | 30.1 | Saccharide |  |  |  | N |
|  | 35.1 | Saccharide | Lipopolysaccharide | Saccharide | 80% | Y |
|  | 36.1 | Saccharide |  |  |  | N |
|  | 36.2 | NRPS* | Turnerbactin | NRP | 38% | N |
|  | 36.3 | Saccharide |  |  |  | N |
|  | 36.4 | Saccharide |  |  |  | N |
|  | 47.1 | Fatty acid |  |  |  | Y |
|  | 54.1 | Terpene* | Carotenoid | Terpene | 100% | N |
|  | 54.2 | Saccharide  Acyl amino acids* | Pyoverdin | NRP | 1% | Y |
|  | 58.1 | Saccharide |  |  |  | Y |
|  | 60.1 | Saccharide |  |  |  | N |
|  | 80.1 | Lassopeptide* |  |  |  | Y |
|  | 93.1 | Saccharide | Emulsan | Saccharide | 9% | Y |
|  | 98.1 | Thiopeptide* | O-antigen | Saccharide | 14% | N |
|  | 98.2 | Saccharide | Taxlllaid A | NRP | 15% | N |
|  | 98.3 | Hserlactone* |  |  |  | N |
|  | 100.1 | Saccharide | Polysaccharide B | Saccharide | 6% | N |
|  | 110.1 | Fatty acid |  |  |  | Y |
|  | 110.2 | Saccharide | Stewartan | Saccharide | 78% | Y |
|  |  |  |  |  |  |  |
| *P. eucalypti* |  |  |  |  |  |  |
| 299R | 18.1 | Saccharide | Emulsan | Saccharide | 9% | N |
| (17 hits) | 19.1 | Saccharide | Lipopolysaccharide | Saccharide | 27% | N |
|  | 21.1 | Saccharide | Polysaccharide B | Saccharide | 6% | N |
|  | 21.2 | Saccharide |  |  |  | N |
|  | 28.1 | Saccharide |  |  |  | N |
|  | 29.1 | Siderophore* | Desferrioxamine E | Other | 100% | N |
|  | 29.2 | Terpene* | Carotenoid | Terpene | 100% | N |
|  | 30.1 | Saccharide |  |  |  | N |
|  | 30.2 | Saccharide |  |  |  | N |
|  | 31.1 | Thiopeptide* | O-antigen | Saccharide | 14% | N |
|  | 31.2 | Saccharide | Taxlllaid A | NRP | 13% | N |
|  | 31.3 | Hserlactone*  Arylpolyene*  Saccharide | Aryl polyenes | Other | 88% | N |
|  | 31.4 | Fatty acid |  |  |  | N |
|  | 31.5 | Saccharide |  |  |  | N |
|  | 32.1 | Saccharide |  |  |  | N |
|  | 35.1 | Fatty acid |  |  |  | N |
|  | 35.2 | Saccharide | Stewartan | Saccharide | 92% | N |
|  |  |  |  |  |  |  |
| B011489 | 1.1 | Saccharide |  |  |  | N |
| (18 hits) | 2.1 | Saccharide | Emulsan | Saccharide | 9% | N |
|  | 4.1 | Saccharide |  |  |  | N |
|  | 4.2 | Fatty acid |  |  |  | N |
|  | 4.3 | Arylpolyene*  Saccharide  Hserlactone* | Aryl polyenes | Other | 88% | N |
|  | 4.4 | Saccharide | Taxlllaid A | NRP | 13% | N |
|  | 4.5 | Thiopeptide* | O-antigen | Saccharide | 14% | N |
|  | 5.1 | Saccharide | O&K-antigen | Saccharide | 4% | N |
|  | 8.1 | Siderophore* | Desferrioxamine E | Other | 100% | N |
|  | 8.2 | Terpene* | Carotenoid | Terpene | 100% | N |
|  | 8.3 | Siderophore* | Desferrioxamine E | Other | 100% | N |
|  | 8.4 | Terpene* | Carotenoid | Terpene | 100% | N |
|  | 18.1 | Saccharide | Polysaccharide B | Saccharide | 6% | N |
|  | 18.2 | Saccharide |  |  |  | N |
|  | 24.1 | Saccharide |  |  |  | N |
|  | 25.1 | Saccharide |  |  |  | N |
|  | 26.1 | Saccharide | Stewartan | Saccharide | 92% | N |
|  | 26.2 | Fatty acid |  |  |  | N |
|  |  |  |  |  |  |  |
| F9026  (17 hits) | 2.1 | Saccharide  Fatty acid |  |  |  | N |
|  | 7.1 | Saccharide | Emulsan | Saccharide | 9% | N |
|  | 12.1 | Saccharide |  |  |  | N |
|  | 14.1 | Siderophore* | Desferrioxamine E | Other | 100% | N |
|  | 14.2 | Terpene* | Carotenoid | Terpene | 100% | N |
|  | 17.1 | Saccharide | O&K-antigen | Saccharide | 4% | N |
|  | 18.1 | Saccharide |  |  |  | N |
|  | 19.1 | Saccharide |  |  |  | N |
|  | 19.2 | Saccharide | Polysaccharide B | Saccharide | 6% | N |
|  | 23.1 | Thiopeptide* | O-antigen | Saccharide | 14% | N |
|  | 23.2 | Saccharide | Taxlllaid A | NRP | 13% | N |
|  | 23.3 | Hserlactone*  Arylpolyene*  Saccharide | Aryl polyenes | Other | 88% | N |
|  | 23.4 | Fatty acid |  |  |  | N |
|  | 23.5 | Saccharide |  |  |  | N |
|  | 26.1 | Saccharide | Stewartan | Saccharide | 92% | N |
|  | 26.2 | Fatty acid |  |  |  | N |
|  | 28.1 | Saccharide |  |  |  | N |
|  |  |  |  |  |  |  |
| SP02021  (19 hits) | 1.1 | Arylpolyene*  Saccharide | Aryl polyenes | Other | 88% | N |
|  | 1.2 | Fatty acid |  |  |  | N |
|  | 1.3 | Saccharide |  |  |  | N |
|  | 3.1 | Fatty acid |  |  |  | N |
|  | 5.1 | Thiopeptide* | O-antigen | Saccharide | 14% | N |
|  | 5.2 | Saccharide | Taxlllaid A | NRP | 13% | N |
|  | 5.3 | Hserlactone* |  |  |  | N |
|  | 6.1 | Saccharide |  |  |  | N |
|  | 8.1 | Saccharide | Stewartan | Saccharide | 92% | N |
|  | 10.1 | Terpene* | Carotenoid | Terpene | 100% | N |
|  | 10.2 | Siderophore* | Desferrioxamine E | Other | 100% | N |
|  | 13.1 | Saccharide | O&K-antigen | Saccharide | 4% | N |
|  | 15.1 | Saccharide |  |  |  | N |
|  | 16.1 | Fatty acid  Saccharide |  |  |  | N |
|  | 20.1 | Saccharide |  |  |  | N |
|  | 21.1 | Saccharide |  |  |  | N |
|  | 22.1 | Saccharide | Emulsan | Saccharide | 9% | N |
|  | 25.1 | Saccharide |  |  |  | N |
|  | 25.2 | Saccharide | Polysaccharide B | Saccharide | 6% | N |
|  |  |  |  |  |  |  |
| SP03372 | 4.1 | Saccharide | Emulsan | Saccharide | 9% | N |
| (18 hits) | 4.2 | Saccharide |  |  |  | N |
|  | 9.1 | Terpene* | Carotenoid | Terpene | 100% | N |
|  | 9.2 | Siderophore* | Desferrioxamine E | Other | 100% | N |
|  | 16.1 | Saccharide |  |  |  | N |
|  | 20.1 | Saccharide | O&K-antigen | Saccharide | 4% | N |
|  | 21.1 | Saccharide |  |  |  | N |
|  | 22.1 | Saccharide |  |  |  | N |
|  | 22.2 | Fatty acid |  |  |  | N |
|  | 22.3 | Arylpolyene*  Saccharide  Hserlactone* | Aryl polyenes | Other | 88% | N |
|  | 22.4 | Saccharide | Taxlllaid A | NRP | 13% | N |
|  | 22.5 | Thiopeptide* | O-antigen | Saccharide | 14% | N |
|  | 23.1 | NRPS* |  |  |  | N |
|  | 23.2 | Saccharide |  |  |  | N |
|  | 23.3 | Saccharide | Polysaccharide B | Saccharide | 6% | N |
|  | 23.4 | Saccharide | Stewartan | Saccharide | 92% | N |
|  | 23.5 | Fatty acid |  |  |  | N |
|  | 24.1 | Saccharide |  |  |  | N |
|  |  |  |  |  |  |  |
| SP03391  (21 hits) | 2.1 | Saccharide  Fatty acid | O-antigen | Saccharide | 10% | N |
|  | 6.1 | Saccharide |  |  |  | N |
|  | 10.1 | Saccharide |  |  |  | N |
|  | 15.1 | Siderophore* | Desferrioxamine E | Other | 100% | N |
|  | 15.2 | Terpene* | Carotenoid | Terpene | 100% | N |
|  | 15.3 | Siderophore* | Desferrioxamine E | Other | 100% | N |
|  | 15.4 | Terpene* | Carotenoid | Terpene | 100% | N |
|  | 16.1 | Saccharide | Stewartan | Saccharide | 92% | N |
|  | 16.2 | Fatty acid |  |  |  | N |
|  | 17.1 | Thiopeptide* | O-antigen | Saccharide | 14% | N |
|  | 17.2 | Saccharide | Taxlllaid A | NRP | 13% | N |
|  | 17.3 | Hserlactone* |  |  |  | N |
|  | 17.4 | Arylpolyene*  Saccharide | Aryl polyenes | Other | 88% | N |
|  | 17.5 | Fatty acid |  |  |  | N |
|  | 17.6 | Saccharide |  |  |  | N |
|  | 20.1 | Saccharide | O&K-antigen | Saccharide | 4% | N |
|  | 22.1 | Saccharide | Emulsan | Saccharide | 9% | N |
|  | 26.1 | Saccharide | Polysaccharide B | Saccharide | 6% | N |
|  | 26.2 | Saccharide |  |  |  | N |
|  | 26.3 | Saccharide |  |  |  | N |
|  | 28.1 | Saccharide |  |  |  | N |
|  |  |  |  |  |  |  |
| SP04013 | 1.1 | Saccharide | Polysaccharide B | Saccharide | 6% | N |
| (16 hits) | 1.2 | Saccharide |  |  |  | N |
|  | 5.1 | Saccharide |  |  |  | N |
|  | 6.1 | Saccharide |  |  |  | N |
|  | 9.1 | Terpene* | Carotenoid | Terpene | 100% | N |
|  | 9.2 | Siderophore* | Desferrioxamine E | Other | 100% | N |
|  | 11.1 | Saccharide | Emulsan | Saccharide | 9% | N |
|  | 13.1 | Saccharide | Lipopolysaccharide | Saccharide | 27% | N |
|  | 18.1 | Saccharide |  |  |  | N |
|  | 23.1 | Saccharide |  |  |  | N |
|  | 23.2 | Fatty acid |  |  |  | N |
|  | 23.3 | Arylpolyene*  Saccharide  Hserlactone* | Aryl polyenes | Other | 88% | N |
|  | 23.4 | Saccharide | Taxlllaid A | NRP | 13% | N |
|  | 23.5 | Thiopeptide* | O-antigen | Saccharide | 14% | N |
|  | 25.1 | Saccharide | Stewartan | Saccharide | 92% | N |
|  | 25.2 | Fatty acid |  |  |  | N |
|  |  |  |  |  |  |  |
| *P. septica* |  |  |  |  |  |  |
| VB38951A | 54.1 | Terpene* | Carotenoid | Terpene | 100% | Y |
| (19 hits) | 65.1 | Thiopeptide* | O-antigen | Saccharide | 14% | N |
|  | 69.1 | NRPS* |  |  |  | Y |
|  | 71.1 | Saccharide |  |  |  | N |
|  | 72.1 | Saccharide |  |  |  | Y |
|  | 75.1 | Fatty acid |  |  |  | Y |
|  | 112.1 | Saccharide | O&K-antigen | Saccharide | 3% | N |
|  | 115.1 | Saccharide |  |  |  | Y |
|  | 125.1 | Saccharide |  |  |  | Y |
|  | 130.1 | Saccharide |  |  |  | Y |
|  | 132.1 | Siderophore* | Aerobactin | Other | 66% | N |
|  | 134.1 | Saccharide | Polysaccharide B | Saccharide | 6% | N |
|  | 134.2 | Saccharide |  |  |  | Y |
|  | 136.1 | Saccharide | Stewartan | Saccharide | 92% | N |
|  | 138.1 | Saccharide | O&K-antigen | Saccharide | 4% | N |
|  | 143.1 | Fatty acid |  |  |  | Y |
|  | 143.2 | Hserlactone* |  |  |  | N |
|  | 143.3 | Saccharide | Taxlllaid A | NRP | 13% | Y |
|  | 144.1 | Saccharide |  |  |  | N |
|  |  |  |  |  |  |  |
| X44686 | 9.1 | Thiopeptide* | O-antigen | Saccharide | 14% | N |
| (19 hits) | 9.2 | Saccharide | Taxlllaid A | NRP | 13% | N |
|  | 10.1 | Saccharide |  |  |  | N |
|  | 14.1 | Saccharide |  |  |  | N |
|  | 21.1 | Siderophore* | Aerobactin | Other | 66% | Y |
|  | 28.1 | Saccharide |  |  |  | N |
|  | 29.1 | Fatty acid |  |  |  | N |
|  | 30.1 | Hserlactone*  Arylpolyene*  Saccharide | Aryl polyenes | Other | 94% | N |
|  | 30.2 | Fatty acid |  |  |  | Y |
|  | 31.1 | Saccharide | Stewartan | Saccharide | 92% | N |
|  | 42.1 | NRPS* | Turnerbactin | NRP | 38% | N |
|  | 58.1 | Terpene* | Carotenoid | Terpene | 100% | N |
|  | 59.1 | Saccharide |  |  |  | Y |
|  | 63.1 | Saccharide |  |  |  | Y |
|  | 64.1 | Saccharide | O&K-antigen | Saccharide | 3% | N |
|  | 78.1 | Saccharide | O&K-antigen | Saccharide | 4% | N |
|  | 83.1 | Saccharide |  |  |  | N |
|  | 83.2 | Saccharide | Polysaccharide B | Saccharide | 6% | N |
|  | 88.1 | Saccharide |  |  |  | Y |
|  |  |  |  |  |  |  |
| *P. stewartii* |  |  |  |  |  |  |
| 626 | 1.1 | Thiopeptide* | O-antigen | Saccharide | 14% | N |
| (20 hits) | 1.2 | Saccharide | Taxlllaid A | NRP | 13% | N |
|  | 1.3 | Hserlactone* |  |  |  | N |
|  | 1.4 | Fatty acid |  |  |  | N |
|  | 1.5 | Saccharide |  |  |  | N |
|  | 1.6 | Hserlactone* |  |  |  | N |
|  | 1.7 | Saccharide | Polysaccharide B | Saccharide | 6% | N |
|  | 1.8 | Saccharide | Surfactin | NRP:Lipopeptide | 8% | Y |
|  | 2.1 | Fatty acid |  |  |  | N |
|  | 2.2 | Saccharide | Entolysin | NRP | 8% | N |
|  | 2.3 | Saccharide | Stewartan | Saccharide | 92% | N |
|  | 3.1 | Siderophore* | Aerobactin | Other | 77% | N |
|  | 3.2 | Saccharide | N-tetradecanoyl tyrosine | Other | 6% | N |
|  | 3.3 | Siderophore* | Desferrioxamine E | Other | 100% | N |
|  | 3.4 | Saccharide |  |  |  | N |
|  | 4.1 | Saccharide |  |  |  | N |
|  | 4.2 | Terpene* | Carotenoid | Terpene | 100% | N |
|  | 7.1 | Saccharide | Emulsan | Saccharide | 9% | N |
|  | 8.1 | Saccharide |  |  |  | N |
|  | 11.1 | Saccharide | O&K-antigen | Saccharide | 4% | N |
|  |  |  |  |  |  |  |
| DC283 | 8.1 | Saccharide | Emulsan | Saccharide | 9% | Y |
| (21 hits) | 11.1 | Fatty acid |  |  |  | N |
|  | 16.1 | Thiopeptide* | O-antigen | Saccharide | 14% | N |
|  | 17.1 | Saccharide | Taxlllaid A | NRP | 13% | N |
|  | 23.1 | Fatty acid | Colicin V | RiPP | 1% | Y |
|  | 32.1 | Saccharide | O-antigen | Saccharide | 9% | Y |
|  | 39.1 | Saccharide | Lipopolysaccharide | Saccharide | 40% | Y |
|  | 40.1 | Saccharide | O&K-antigen | Saccharide | 3% | Y |
|  | 43.1 | Saccharide | Stewartan | Saccharide | 92% | N |
|  | 53.1 | Saccharide | Colicin V | RiPP | 1% | Y |
|  | 54.1 | Siderophore* | Aerobactin | Other | 77% | N |
|  | 54.2 | Saccharide | Colicin V | RiPP | 1% | N |
|  | 62.1 | Hserlactone* | Colicin V | RiPP | 1% | Y |
|  | 65.1 | Saccharide |  |  |  | N |
|  | 75.1 | Siderophore* | Desferrioxamine E | Other | 50% | Y |
|  | 76.1 | Saccharide |  |  |  | N |
|  | 76.2 | Siderophore* | Desferrioxamine E | Other | 75% | N |
|  | 84.1 | Saccharide |  |  |  | N |
|  | 86.1 | Terpene* | Carotenoid | Terpene | 100% | N |
|  | 93.1 | Saccharide | Colicin V | RiPP | 1% | Y |
|  | 97.1 | Saccharide | Polysaccharide B | Saccharide | 6% | Y |

*hits also identified with ‘relaxed’ detection strictness

Supplementary Table 7. Identification of PNP, pantocin A, and cluster 675 biosynthetic gene clusters by secondary metabolite software.*

| Software | BRT175 PNP-1 | TX10 PNP-2 | SN01080 PNP-3 | 3581 PNP-3 | 3581 pantocin A | B025670 675 |
| --- | --- | --- | --- | --- | --- | --- |
| antiSMASH | N | N | N^a^ | P | N | P |
| BAGEL4 | N | N | N^a^ | N^a^ | N | N |
| NP.searcher | N | N | N | N | N | N |
| PRISM | N | N | N | P | Y | N |

*P, genes identified as part of larger cluster; N, cluster not identified; Y, full cluster identified

^a^Data reported previously (Williams and Stavrinides, 2020)

Supplementary Table 8. Cross-referenced EDGAR trial 1 and antiSMASH hits.*

| EDGAR gene | Description | antiSMASH region | Hit start | Hit end | Region length |
| --- | --- | --- | --- | --- | --- |
| EOANGEPJ_00438 | mannose-1-phosphate guanylyltransferase/mannose-6-phosphate isomerase | 18.1 | 21404 | 22819 | 51747 |
| EOANGEPJ_00439 | phosphomannomutase | 18.1 | 20007 | 21389 | 51747 |
| EOANGEPJ_00440 | GDP mannose 4,6-dehydratase | 18.1 | 18918 | 19973 | 51747 |
| EOANGEPJ_00441 | glycosyl transferase family 1 | 18.1 | 17661 | 18689 | 51747 |
| EOANGEPJ_00442 | ABC transporter permease | 18.1 | 16871 | 17647 | 51747 |
| EOANGEPJ_00444 | mannosyltransferase | 18.1 | 13242 | 16151 | 51747 |
| EOANGEPJ_00445 | GDP-6-deoxy-D-lyxo-4-hexulose reductase | 18.1 | 12324 | 13235 | 51747 |
| EOANGEPJ_00446 | acyltransferase | 18.1 | 11176 | 12324 | 51747 |
| EOANGEPJ_00447 | mannosyltransferase | 18.1 | 10001 | 11158 | 51747 |
| EOANGEPJ_01329 | hypothetical protein | 32.2 | 19595 | 20639 | 20639 |
| EOANGEPJ_01330 | hypothetical protein | 32.2 | 18169 | 19602 | 20639 |
| EOANGEPJ_01331 | restriction endonuclease subunit S | 32.2 | 16802 | 18172 | 20639 |
| EOANGEPJ_01332 | type I restriction-modification protein subunit M | 32.2 | 14349 | 16805 | 20639 |
| EOANGEPJ_01334 | RelE family toxin-antitoxin system | 32.2 | 12405 | 12764 | 20639 |
| EOANGEPJ_01342 | transcriptional regulator | 32.2 | 6277 | 6525 | 20639 |
| EOANGEPJ_02257 | DNA primase | 4.1 | 20266 | 21246 | 21302 |
| EOANGEPJ_02258 | hypothetical protein | 4.1 | 19584 | 20195 | 21302 |
| EOANGEPJ_02259 | hypothetical protein | 4.1 | 19203 | 19511 | 21302 |
| EOANGEPJ_02260 | hypothetical protein | 4.1 | 18776 | 19093 | 21302 |
| EOANGEPJ_02261 | hypothetical protein | 4.1 | 18308 | 18682 | 21302 |
| EOANGEPJ_02262 | putative protein{ECO:0000313\|EMBL:CCF08178.1} | 4.1 | 17804 | 18220 | 21302 |
| EOANGEPJ_02263 | hypothetical protein | 4.1 | 16372 | 17286 | 21302 |
| EOANGEPJ_02265 | 3-oxoacyl-ACP reductase | 4.1 | 12767 | 13540 | 21302 |
| EOANGEPJ_02266 | hypothetical protein | 4.1 | 12373 | 12765 | 21302 |
| EOANGEPJ_02267 | hypothetical protein | 4.1 | 11305 | 12369 | 21302 |
| EOANGEPJ_02268 | beta-ketoacyl-ACP synthase II | 4.1 | 10001 | 11302 | 21302 |
| EOANGEPJ_02269 | AMP-dependent synthetase | 4.1 | 8598 | 10004 | 21302 |
| EOANGEPJ_02270 | hypothetical protein | 4.1 | 7959 | 8594 | 21302 |
| EOANGEPJ_02271 | multidrug efflux pump | 4.1 | 4852 | 7935 | 21302 |
| EOANGEPJ_02272 | methyltransferase domain-containing protein | 4.1 | 4150 | 4815 | 21302 |
| EOANGEPJ_02273 | hypothetical protein | 4.1 | 2954 | 4150 | 21302 |
| EOANGEPJ_02274 | hypothetical protein | 4.1 | 2131 | 2949 | 21302 |
| EOANGEPJ_02275 | 2-methylcitrate synthase | 4.1 | 1080 | 2141 | 21302 |
| EOANGEPJ_02276 | hypothetical protein | 4.1 | 349 | 1071 | 21302 |
| EOANGEPJ_02277 | hypothetical protein | 4.1 | 1 | 336 | 21302 |
| EOANGEPJ_03037 | N-6 DNA methylase | 31.1 | 17919 | 18098 | 22122 |
| EOANGEPJ_03956 | type VI secretion protein | 21.2 | 11927 | 12313 | 20445 |
| EOANGEPJ_03958 | type VI secretion protein | 21.2 | 10805 | 11191 | 20445 |
| EOANGEPJ_04499 | hypothetical protein | 21.2 | 14928 | 15410 | 20445 |
| EOANGEPJ_04501 | lipoprotein | 21.2 | 12396 | 13067 | 20445 |

*colour coded by antiSMASH region; B025670 vs. *P. agglomerans* DC432 and SP04022

Supplementary Table 9. Cross-referenced EDGAR trial 2 and antiSMASH hits.*

| EDGAR gene | Description | antiSMASH region | Hit start | Hit end | Region length |
| --- | --- | --- | --- | --- | --- |
| EOANGEPJ_00442 | ABC transporter permease | 18.1 | 16871 | 17647 | 51747 |
| EOANGEPJ_01329 | hypothetical protein | 32.2 | 19595 | 20639 | 20639 |
| EOANGEPJ_01330 | hypothetical protein | 32.2 | 18169 | 19602 | 20639 |
| EOANGEPJ_01331 | restriction endonuclease subunit S | 32.2 | 16802 | 18172 | 20639 |
| EOANGEPJ_01332 | type I restriction-modification protein subunit M | 32.2 | 14349 | 16805 | 20639 |
| EOANGEPJ_02263 | hypothetical protein | 4.1 | 16372 | 17286 | 21302 |
| EOANGEPJ_02265 | 3-oxoacyl-ACP reductase | 4.1 | 12767 | 13540 | 21302 |
| EOANGEPJ_02266 | hypothetical protein | 4.1 | 12373 | 12765 | 21302 |
| EOANGEPJ_02267 | hypothetical protein | 4.1 | 11305 | 12369 | 21302 |
| EOANGEPJ_02268 | beta-ketoacyl-ACP synthase II | 4.1 | 10001 | 11302 | 21302 |
| EOANGEPJ_02269 | AMP-dependent synthetase | 4.1 | 8598 | 10004 | 21302 |
| EOANGEPJ_02270 | hypothetical protein | 4.1 | 7959 | 8594 | 21302 |
| EOANGEPJ_02271 | multidrug efflux pump | 4.1 | 4852 | 7935 | 21302 |
| EOANGEPJ_02272 | methyltransferase domain-containing protein | 4.1 | 4150 | 4815 | 21302 |
| EOANGEPJ_02273 | hypothetical protein | 4.1 | 2954 | 4150 | 21302 |
| EOANGEPJ_02274 | hypothetical protein | 4.1 | 2131 | 2949 | 21302 |
| EOANGEPJ_02275 | 2-methylcitrate synthase | 4.1 | 1080 | 2141 | 21302 |
| EOANGEPJ_02276 | hypothetical protein | 4.1 | 349 | 1071 | 21302 |
| EOANGEPJ_02277 | hypothetical protein | 4.1 | 1 | 336 | 21302 |
| EOANGEPJ_03956 | type VI secretion protein | 21.2 | 11927 | 12313 | 20445 |
| EOANGEPJ_03958 | type VI secretion protein | 21.2 | 10805 | 11191 | 20445 |
| EOANGEPJ_04499 | hypothetical protein | 21.2 | 14928 | 15410 | 20445 |
| EOANGEPJ_04501 | lipoprotein | 21.2 | 12396 | 13067 | 20445 |

*colour coded by antiSMASH region; B025670 vs. *P. agglomerans* DC432, *P. agglomerans* SP04022, *P. dispersa* 625, *P. stewartii* 626, *P. eucalypti* B011489, *P. brenneri* B016381, *P. brenneri* B024858, *P.* *eucalypti* F9026, *P. ananatis* 15320, *P. ananatis* 17671, and *P. ananatis* 26SR6

**References**

Alexeyev, M. F. (1999). The pKNOCK series of broad-host-range mobilizable suicide vectors for gene knockout and targeted DNA insertion into the chromosome of Gram-negative bacteria. *Biotechniques* 26, 824–828. doi:10.2144/99265bm05.

De Maayer, P., Chan, W. Y., Rezzonico, F., Bühlmann, A., Venter, S. N., Blom, J., et al. (2012). Complete genome sequence of clinical isolate *Pantoea ananatis* LMG 5342. *J. Bacteriol.* 194, 1615–1616. doi:10.1128/JB.06715-11.

De Maayer, P., Chan, W. Y., Venter, S. N., Toth, I. K., Birch, P. R. J., Joubert, F., et al. (2010). Genome sequence of *Pantoea ananatis* LMG20103, the causative agent of *Eucalyptus* blight and dieback. *J. Bacteriol.* 192, 2936–2937. doi:10.1128/JB.00060-10.

Kessler, B., de Lorenzo, V., and Timmis, K. N. (1992). A general system to integrate *lacZ* fusions into the chromosomes of Gram-negative eubacteria: regulation of the *Pm* promoter of the *TOL* plasmid studied with all controlling elements in monocopy. *Mol. Gen. Genet.* 233, 293–301. doi:10.1007/bf00587591.

Nadarasah, G., and Stavrinides, J. (2014). Quantitative evaluation of the host-colonizing capabilities of the enteric bacterium *Pantoea* using plant and insect hosts. *Microbiology* 160, 602–615. doi:10.1099/mic.0.073452-0.

Robinson, L. J., Verrett, J. N., Sorout, N., and Stavrinides, J. (2020). A broad-spectrum antibacterial natural product from the cystic fibrosis isolate, *Pantoea agglomerans* Tx10. *Microbiol. Res.* 237, 126479. doi:10.1016/j.micres.2020.126479.

Soutar, C. D., and Stavrinides, J. (2019). Molecular validation of clinical *Pantoea* isolates identified by MALDI-TOF. *PLoS One* 14, e0224731. doi:10.1371/journal.pone.0224731.

Walterson, A. M., Smith, D. D. N., and Stavrinides, J. (2014). Identification of a *Pantoea* biosynthetic cluster that directs the synthesis of an antimicrobial natural product. *PLoS One* 9, e96208. doi:10.1371/journal.pone.0096208.

Williams, A. N., and Stavrinides, J. (2020). *Pantoea* Natural Product 3 is encoded by an eight-gene biosynthetic gene cluster and exhibits antimicrobial activity against multi-drug resistant *Acinetobacter baumannii* and *Pseudomonas aeruginosa*. *Microbiol. Res.* 234, 126412. doi:10.1016/j.micres.2020.126412.
